# Murine Gamma Herpesvirus 68 Hijacks MAVS and IKKβ to Abrogate NFκB Activation and Antiviral Cytokine Production

**DOI:** 10.1371/journal.ppat.1002336

**Published:** 2011-11-10

**Authors:** Xiaonan Dong, Pinghui Feng

**Affiliations:** 1 Department of Microbiology, University of Texas Southwestern Medical Center, Dallas, Texas, United States of America; 2 Department of Molecular Microbiology and Immunology, University of Southern California, Los Angeles, California, United States of America; University of North Carolina at Chapel Hill, United States of America

## Abstract

Upon viral infection, mitochondrial antiviral signaling (MAVS) protein serves as a key adaptor to promote cytokine production. We report here that murine gamma herpesvirus 68 (γHV68), a model virus for oncogenic human gamma herpesviruses, subverts cytokine production via the MAVS adaptor. During early infection, γHV68 hijacks MAVS and IKKβ to induce the site-specific phosphorylation of RelA, a crucial subunit of the transcriptionally active NFκB dimer, which primes RelA for the proteasome-mediated degradation. As such, γHV68 efficiently abrogated NFκB activation and cytokine gene expression. Conversely, uncoupling RelA degradation from γHV68 infection promoted NFκB activation and elevated cytokine production. Loss of MAVS increased cytokine production and immune cell infiltration in the lungs of γHV68-infected mice. Moreover, exogenous expression of the phosphorylation- and degradation-resistant RelA variant restored γHV68-induced cytokine production. Our findings uncover an intricate strategy whereby signaling *via* the upstream MAVS adaptor is intercepted by a pathogen to nullify the immediate downstream effector, RelA, of the innate immune pathway.

## Author Summary

Innate immunity represents the first line of defense against invading pathogens chiefly through anti-viral cytokines. The mitochondrial antiviral signaling (MAVS)-dependent innate immune pathways are critical for inflammatory cytokine production. Deficiency in essential innate immune components, such as MAVS, severely impairs cytokine production and host defense that are enabled by the master transcription factor, NFκB. Here we show that murine gamma herpesvirus 68 (γHV68), a model herpesvirus for human Kaposi's sarcoma-associated herpesvirus and Epstein-Barr virus, hijacks MAVS and IKKβ to abrogate NFκB activation and cytokine production. Uncoupling RelA degradation from γHV68 infection restored NFκB-dependent cytokine gene expression and elevated cytokine production. Thus, our results demonstrate that upstream innate immune activation can be harnessed by pathogens to inactivate the downstream effector and subvert cytokine production.

## Introduction

Innate immunity represents the first line of defense against invading pathogens. Eukaryotic cells express a panel of sensors, known as pattern recognition receptors (PRRs), which detect pathogen-associated molecular patterns that are either structural components or replication intermediates [1], [2]. Toll-like receptors are primarily expressed on immune cells and patrol the extracellular and endosomal compartments. The recently discovered cytosolic receptors (e.g., NOD-like receptors and RIG-I-like receptors) are more ubiquitously expressed and monitor the presence of pathogens in the cytosol. Along with C-type lectins [3], these sentinel molecules constitute the vast majority of PRRs in high eukaryotes.

The cytosolic RIG-I and MDA-5 sensors are authentic RNA helicases that contain two tandem **ca**spase-**r**ecruitment **d**omains (CARD) within the amino-terminus and an RNA-binding domain within the carboxyl terminus, endowing the ability to detect nucleic acids [4], [5]. Association with RNA triggers the dimerization of RIG-I and MDA-5 with the **m**itochondrial **a**nti**v**iral **s**ignaling (MAVS, also known as IPS-1, VISA, and CARDIF) adaptor via their N-terminal CARDs, which relays signal to promote antiviral cytokine production [6], [7], [8], [9]. In doing so, MAVS activates the IKKα/β/γ and TBK1/IKKε kinase complexes that, through phosphorylation, effectively promote the gene expression driven by transcription factors of the NFκB and interferon regulatory factor (IRF) family, respectively [10], [11], [12], [13]. It is believed that NFκB activation sufficiently induces the expression of inflammatory cytokines, such as IL6 and TNFα. The efficient transcriptional activation of a prototype interferon (IFN), IFN-β, requires the concerted action of multiple transcription factors including NFκB, ATF2, c-Jun, and IRFs, constituting one of the most sophisticated coordination within multiple innate immune signaling pathways to achieve optimal antiviral immune responses [14], [15]. The participation of numerous components in relaying signaling from pathogen detection to cytokine production maximizes the number of checkpoints to tune host immune responses. Conversely, the highly ordered architecture of signaling cascades also offers pathogens with opportunities to manipulate and exploit host immune responses. Key to the immune signaling cascades is the activation of NFκB transcription factors that control cytokine production, an essential determinant underlying effective host innate and adaptive immune responses.

The family of NFκB transcription factors is composed of five members, including RelA (p65), RelB, c-Rel, NFκB1 (p50 derived from its precursor p100), and NFκB2 (p52 derived from its precursor p105) [16]. All NFκB transcription factors share an N-terminal Rel homology domain that is responsible for subunit dimerization and sequence-specific DNA binding activity. Additionally, RelA, RelB, and c-Rel harbor a C-terminal transcription activation domain (TAD) that positively regulates gene transcription. Among them, RelA is the most ubiquitously and abundantly expressed subunit. By contrast, NFκB1 and NFκB2 do not contain a TAD and therefore rely on dimerization with one of the other three NFκB members to activate gene transcription. Furthermore, post-translational modifications, such as phosphorylation and acetylation, have been identified to confer specific effect on the DNA-binding, protein stability, and transcriptional activity of NFκB transcription factors [17], [18]. Although the signaling pathways that activate NFκB transcription factors have been extensively investigated, relatively little is known regarding the equally important process of NFκB termination.

Herpesviruses are large DNA viruses that establish a lifelong persistent infection. To persist within immuno-competent hosts, gamma herpesviruses in particular have evolved an arsenal of weapons to contend with host immune responses [19], [20]. Being closely-related to human oncogenic Kaposi's sarcoma-associated herpesvirus (KSHV) and Epstein-Barr virus (EBV), murine gamma herpesvirus 68 (γHV68) infects laboratory strains of mice, resulting in robust acute infection in the lung and persistent infection in the spleen. Thus, murine infection with γHV68 offers a tractable small animal model to examine the entire course of host immune responses and viral infection *in vivo*, which are not available for human KSHV and EBV [21].

To assess the role of immune signaling pathways downstream of cytosolic sensors in gamma herpesvirus infection, we have characterized viral infection and host innate immune responses in MAVS-deficient mice infected with γHV68. We previously reported that γHV68 activates the MAVS-IKKβ pathway to promote viral lytic infection [22]. Paradoxically, the activation of the MAVS-IKKβ pathway often instigates NFκB activation and the production of antiviral cytokines [23], [24]. Moreover, RelA was shown to inhibit γHV68 lytic replication [25]. We report here that γHV68 utilizes MAVS and IKKβ to promote RelA phosphorylation and transient degradation, thereby efficiently abrogating NFκB activation and cytokine production. Finally, loss of MAVS elevated inflammatory cytokines and immune cell infiltration in the lung of γHV68-infected mice, highlighting an essential role of MAVS in evading antiviral cytokine production. Our findings illustrate an intricate strategy whereby a pathogen usurps upstream immune signaling events of the NFκB pathway to destroy the essential downstream effector, RelA, effectively nullifying host innate cytokine production. These findings reshape our view on host innate immune responses.

## Results

### Loss of MAVS Results in Increased Cytokine Production in Response to γHV68 Infection

**Figure 1:**
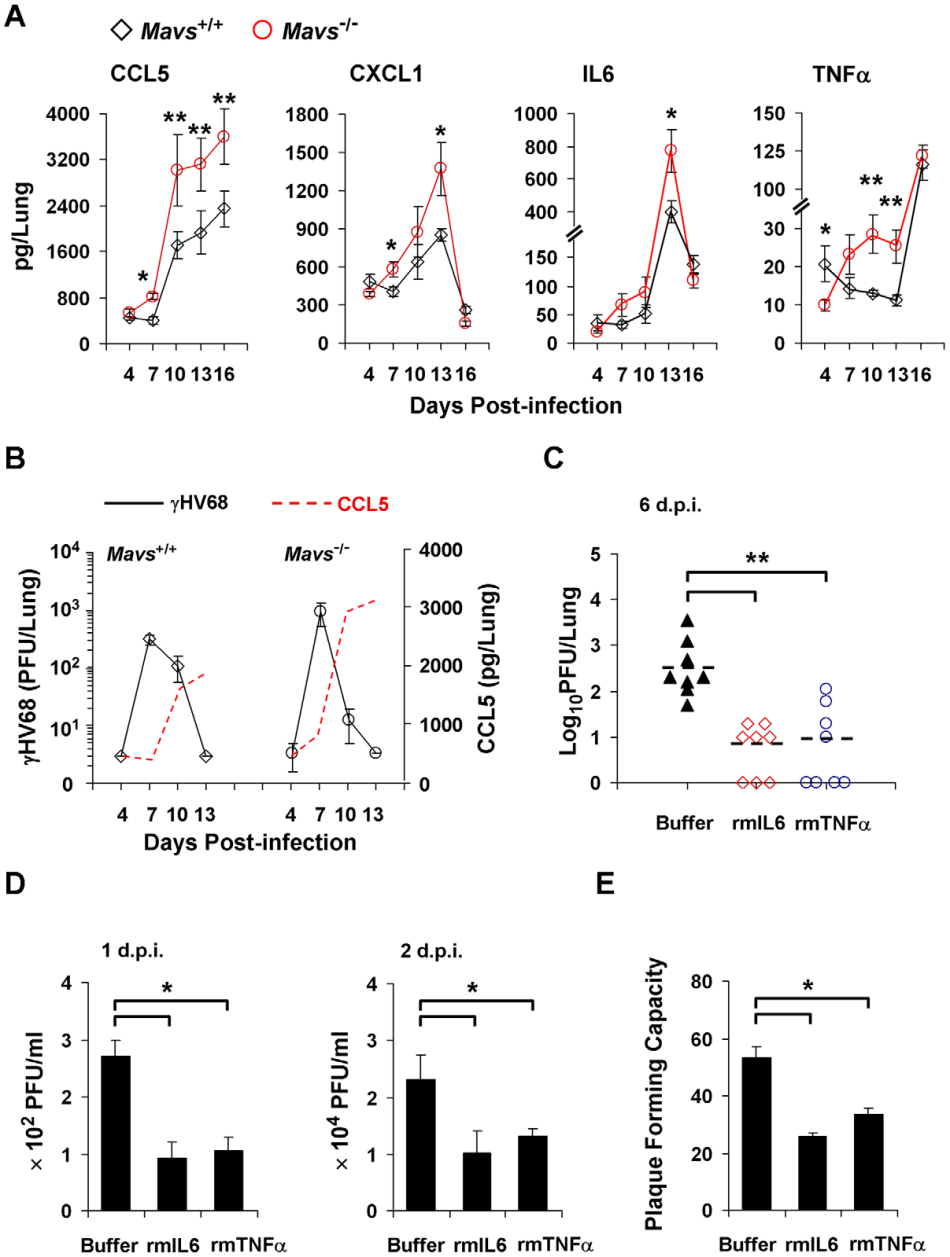
MAVS deficiency increases inflammatory cytokines in the lung of γHV68-infected mice. (A and B) Age- and gender-matched *Mavs* ^+/+^ and *Mavs* ^−/−^ littermate mice (n = 8) were intranasally infected with 40 plaque-forming units (PFU) of γHV68. (A) Increased inflammatory cytokines in the lung of γHV68-infected *Mavs* ^−/−^ mice compared with *Mavs* ^+/+^ mice. (B) γHV68 loads in the lung of infected mice were determined by a plaque assay and shown in comparison to CCL5 levels. Data are presented as the mean ± the standard error of the mean (SEM) of eight mice. See also Figure S1. (C) Age- and gender-matched BL6 mice (n = 8) were intranasally infected with 40 PFU of γHV68. Saline buffer, recombinant mouse IL6 or TNFα (rmIL6 or rmTNFα, 30 ng/mouse/day) was intranasally administered from 1 to 5 days post-infection (d.p.i.). Viral loads in the lung were determined by a plaque assay at 6 d.p.i. See also Figure S2 and Figure S3. (D and E) Wild-type mouse embryonic fibroblasts (MEFs) were infected with γHV68 at a multiplicity-of-infection (MOI) of 0.01 (D) or 0.005 (E), with or without rmIL6 or rmTNFα treatment (2 ng/ml, 4 hours). (D) Viral titer in the supernatant collected at 1 d.p.i. and 2 d.p.i. was determined by a plaque assay. (E) At 4 hours post-infection, supernatant was replaced with fresh DMEM containing 2% FBS and 0.75% methylcellulose. Plaques formed in MEF monolayers were counted at 6 d.p.i. Data in (D) and (E) are presented as the mean ± SEM of three independent experiments. The statistical significance in (A), (C), (D) and (E): *, *P*<0.05; **, *P*<0.02.

We have previously shown that γHV68 loads in the lung of *Mavs* ^−/−^ mice were significantly lower than those in the lung of *Mavs* ^+/+^ mice at 10 d.p.i. [22]. We reasoned that the reduced γHV68 acute infection may be, at least partly, due to an elevated immune response in *Mavs* ^−/−^ mice. To test this hypothesis, we measured inflammatory cytokines, including CCL5, CXCL1, IL6 and TNFα in *Mavs* ^+/+^ and *Mavs* ^−/−^ littermate mice infected with a low-dose (40 plaque-forming units, PFU) γHV68 by enzyme-linked immunosorbent assay (ELISA). A low dose infection more likely resembles natural γHV68 infection. We found that, in response to γHV68 infection, levels of all four cytokines in the lung of *Mavs* ^−/−^ mice were approximately two-fold of those in the lung of *Mavs* ^+/+^ mice at 7, 10, and 13 d.p.i. (Figure 1A). This phenomenon is in stark contrast to the observations that loss of MAVS impairs cytokine production in response to viral infection, e.g., RNA viruses [23], [24]. It is notable that γHV68 infection induced significant cytokines in *Mavs* ^−/−^ mice at 7 d.p.i., when cytokines were slightly reduced or unchanged in *Mavs* ^+/+^ mice, indicating a faster cytokine production. Interestingly, cytokine levels in the sera of γHV68-infected *Mavs* ^+/+^ and *Mavs* ^−/−^ mice were similar (Figure S1A). There was no statistically significant difference of IL10, an important anti-inflammatory cytokine, in either lungs or sera of γHV68-infected *Mavs* ^+/+^ and *Mavs* ^−/−^ mice (Figures S1A, S1B). Consistent with our previous reported result [22], loss of MAVS greatly reduced γHV68 load at 10 d.p.i. in the lung, whereas had a marginal effect on viral load at 7 d.p.i. (Figure 1B). These results collectively indicate that, in response to γHV68 infection, *Mavs* ^−/−^ mice produce more inflammatory cytokines specifically in the lung than *Mavs* ^+/+^ mice.

It was reported that γHV68 replicates to similar levels in IL6-deficient and wild-type mice, implying that IL6 is not obligate to limit γHV68 lytic replication [26]. However, our study suggests that γHV68 successfully abrogates cytokine production during early viral infection, the critical stage for cytokines to curtail viral replication. As such, we reasoned that obliterating IL6 does not enhance γHV68 lytic replication, and that the administration of exogenous cytokines likely better evaluates the effect of cytokines on γHV68 acute infection. Thus, we intranasally administered recombinant mouse IL6 or TNFα (rmIL6 or rmTNFα) after a low-dose (40 PFU/mouse) γHV68 infection. We determined that the optimal efficiency of intranasal delivery was approximately 60% (Figure S2). Treatment with either rmIL6 or rmTNFα reduced γHV68 loads in the lung to less than 5% of those in mock-treated mice, demonstrating the potent antiviral effect of rmIL6 and rmTNFα against γHV68 (Figure 1C). Importantly, we found that cytokine treatment did not affect mouse body weight (Figure S3A), spleen mass (Figure S3B), or lung cytokine levels (Figures S3C,S3D), excluding the potential side effect brought by rmIL6 and rmTNFα treatment. Furthermore, we determined whether rmIL6 and rmTNFα inhibit γHV68 lytic replication *ex vivo* under normal productive infection and restricted condition (in methylcellulose-containing medium). Under both conditions, treatment with rmIL6 and rmTNFα reduced γHV68 yield by 60% (Figure 1D) and plaque-forming units by 50% (Figure 1E). Collectively, these results bolster the conclusion that inflammatory cytokines, such as IL6 and TNFα, are potent antiviral effectors against γHV68 lytic replication *ex vivo* and *in vivo*.

### Loss of MAVS Elevates Lung Immune Cell Infiltration in γHV68-infected Mice

**Figure 2:**
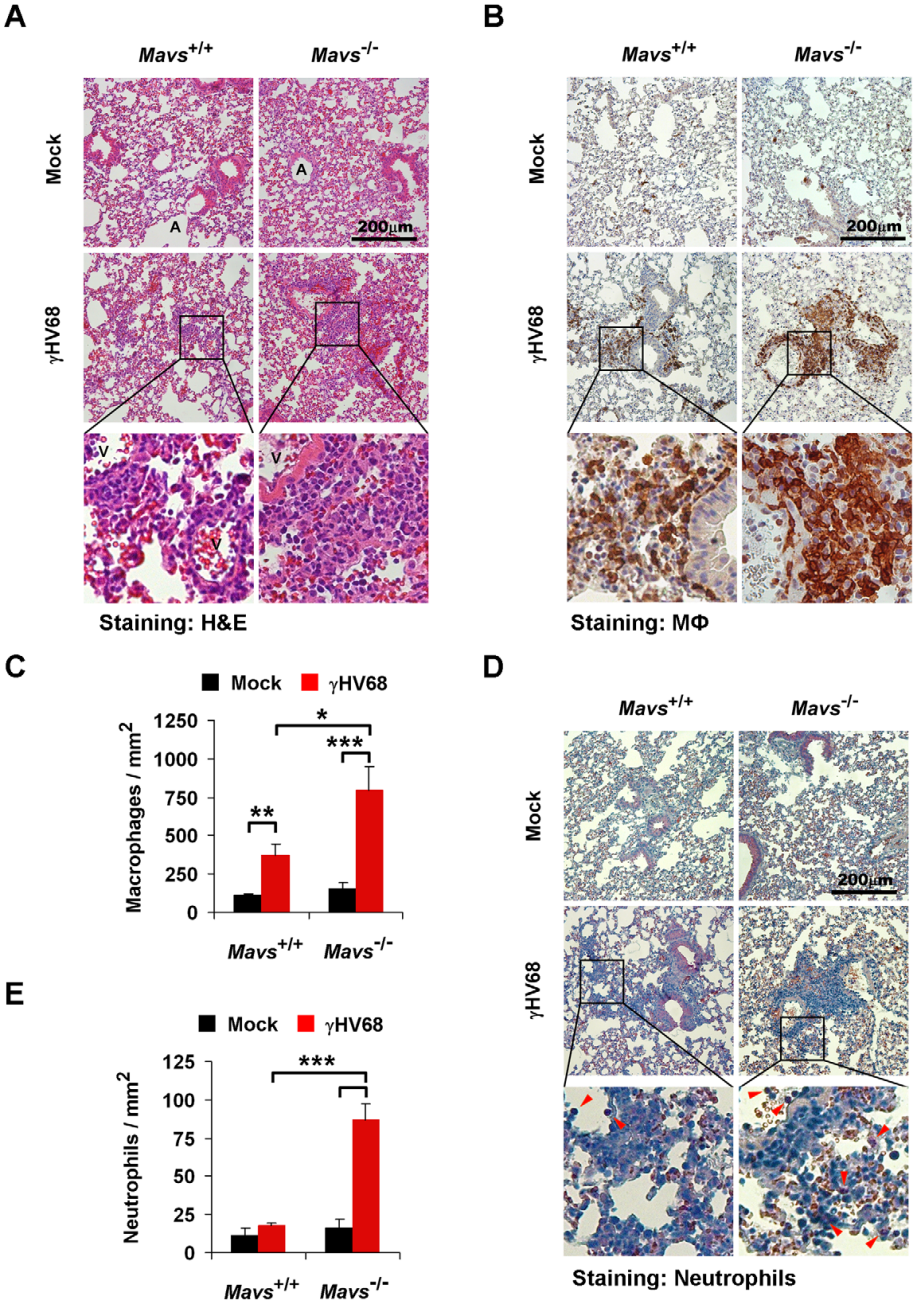
Loss of MAVS increases immune cell infiltration in the lung of γHV68-infected mice. Age- and gender-matched *Mavs* ^+/+^ and *Mavs* ^−/−^ littermate mice were intranasally infected with 40 PFU of γHV68 as in Figure 1A. At 10 days post-infection, mouse lungs were fixed and embedded in paraffin. Three-micrometer paraffin sections of lungs were stained, and pictures were taken at the magnification of 200. One to three optical fields are presented for each group. See also Figure S4. (A) Hematoxylin and eosin (H&E) staining demonstrated a mild mixed-cell infiltration in the lung of γHV68-infected *Mavs* ^+/+^ mice, and an intense peribronchial and perivascular immune cell infiltration in the lung of γHV68-infected *Mavs* ^−/−^ mice. A, airway; V, blood vessel. (B and C) Pulmonary macrophages (*brown*) were probed with anti-Iba1 antibody. (D and E) Pulmonary neutrophils (*red arrowheads*) were selectively stained by an esterase specific assay. In (C) and (E), the number of pulmonary macrophages (C) or perivascular neutrophils (E) was determined by counting eight randomly selected optical fields. Data are presented as the mean ± SEM. The statistical significance: *, *P*<0.05; **, *P*<0.02; ***, *P*<0.005.

Given that CCL5 and CXCL1 represent chemokines important for immune cell recruitment [27], [28], we surmised that the increased levels of CCL5 and CXCL1 in the lung (Figure 1A) likely translate into more robust infiltration of immune cells in *Mavs* ^−/−^ mice than in *Mavs* ^+/+^ littermates. We examined mouse lungs from mock- or γHV68-infected (40 PFU, intranasally) mice by hematoxylin and eosin (H&E) staining. We observed similar lung architecture and no immune cell infiltration in the lung of mock-infected *Mavs* ^+/+^ and *Mavs* ^−/−^ mice (Figures 2A,S4A; top panels). Compared to mock-infected mice, the lungs of both *Mavs* ^+/+^ and *Mavs* ^−/−^ mice at 10 d.p.i. displayed apparent increase in cellularity (Figures 2A,S4A). In the lung of γHV68-infected *Mavs* ^+/+^ mice, there was an isolated mild mixed-cell perivascular infiltration of lymphocytes, macrophages, and rare neutrophils. Strikingly, we observed an intensely increased cellularity within perivascular and peribrochial regions, caused by a massive influx of macrophages, neutrophils, and lymphocytes in the lung of γHV68-infected *Mavs* ^−/−^ mice. Evidently, the immune infiltrated regions extended from blood vessels and bronchioles into alveolar interstitium. It is important to note that, at 7 d.p.i., no significant immune cell infiltration was observed in the lung of either *Mavs* ^+/+^ or *Mavs* ^−/−^ mice (data not shown), suggesting that immune cell infiltration is the consequence of the rising cytokine levels in *Mavs* ^−/−^ lungs at 7 d.p.i. Thus, the expansion of immune infiltrated regions in *Mavs* ^−/−^ lungs is likely the sequela of γHV68 infection and cytokine production thereof.

To further characterize the infiltrated immune cells, pulmonary macrophages were examined by immunohistochemistry staining using a specific antibody, anti-Iba1 (Figure S4B). We observed sporadic and evenly distributed Iba1-positive cells, likely lung-residential macrophages, in mock-infected mice (Figures 2B,S4C; top panels). Consistent with H&E staining, there were much more and larger Iba1-positive foci, in the lung of γHV68-infected *Mavs* ^−/−^ mice than those of *Mavs* ^+/+^ littermates (Figures 2B,S4C), indicative of escalated inflammation in *Mavs* ^−/−^ mice. Moreover, we counted macrophages within eight randomly selected fields, and found that γHV68 infection increased lung macrophages by approximately three-fold in *Mavs* ^+/+^ mice, whereas by more than five-fold in *Mavs* ^−/−^ mice (Figure 2C). Because of the intense staining of Iba1-positive macrophages in *Mavs* ^−/−^ lungs, the increase of macrophages caused by γHV68 infection is likely underestimated.

Neutrophils serve as a hallmark indicator for inflammation and CXCL1 is a major chemokine for neutrophil recruitment. We then examined neutrophils in the lungs of γHV68-infected mice by an esterase specific staining. In contrast to macrophages, neutrophils were rarely observed in the lung of mock-infected mice, or those of γHV68-infected *Mavs* ^+/+^ mice (Figures 2D,S4D). However, neutrophils were easily detected within immune infiltrated regions in the lung of γHV68-infected *Mavs* ^−/−^ mice (Figures 2D,S4D). Counting neutrophils within eight randomly selected fields revealed that γHV68 infection increased neutrophils by approximately five-fold in the lung of *Mavs* ^−/−^ mice, whereas it had a negligible effect on neutrophil recruitment in *Mavs* ^+/+^ mice (Figure 2E). Taken together, these results suggest that the elevated chemokine production promotes the infiltration of immune cells, including macrophages and neutrophils, into γHV68-infected lungs. Altogether, we conclude that MAVS is necessary for γHV68 to dampen cytokine production and subsequent inflammatory responses in the lung.

### γHV68 Abrogates Cytokine Production in a MAVS-dependent Manner

**Figure 3:**
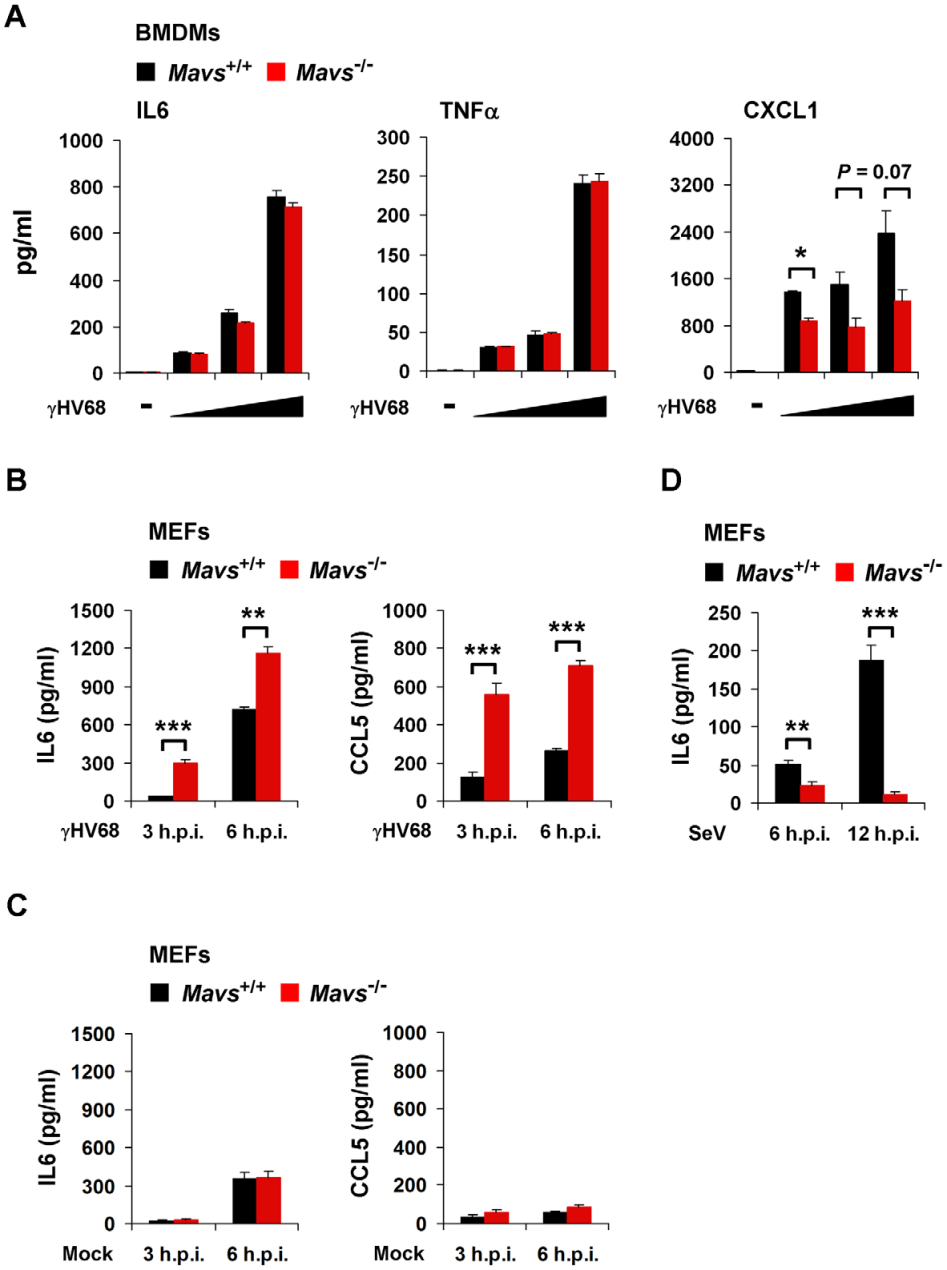
MAVS deficiency results in increased cytokine production in γHV68-infected MEFs. *Mavs* ^+/+^ and *Mavs* ^−/−^ bone marrow-derived macrophages (BMDMs) were infected with γHV68 at an MOI of 0.2, 1, and 5 for 3 hours (A). *Mavs* ^+/+^ and *Mavs* ^−/−^ mouse embryonic fibroblasts (MEFs) were infected with γHV68 at an MOI of 5 (B) or 500 HA units of Sendai virus (SeV) (D), or mock infected (C). Cytokine levels in the supernatant were determined by ELISA. See also Figure S5. (A) IL6, TNFα, and CXCL1 secreted by γHV68-infected *Mavs* ^+/+^ and *Mavs* ^−/−^ BMDMs. (B) IL6 and CCL5 secreted by γHV68-infected *Mavs* ^+/+^ and *Mavs* ^−/−^ MEFs. (C) IL6 and CCL5 secreted by mock-infected *Mavs* ^+/+^ and *Mavs* ^−/−^ MEFs. (D) IL6 secreted by SeV-infected *Mavs* ^+/+^ and *Mavs* ^−/−^ MEFs. Data are presented as the mean ± SEM of three independent experiments. The statistical significance: *, *P*<0.05; **, *P*<0.02; ***, *P*<0.005.

Professional innate immune cells, such as macrophages, are the major source to produce antiviral cytokines and MAVS is crucial for cytokine secretion from macrophages induced by intracellular pathogens [23], [24]. To test whether MAVS deficiency increases cytokine production in γHV68-infected macrophages, we isolated bone marrow-derived macrophages (BMDMs) and determined cytokines secreted by BMDMs in response to γHV68 infection. As expected, loss of MAVS greatly impaired IL6 and CXCL1 secretion in BMDMs infected by Sendai virus (SeV), a prototype RNA virus (Figure S5). Interestingly, we found that γHV68 infection induced equivalent levels of IL6 and TNFα in *Mavs* ^+/+^ and *Mavs* ^−/−^ BMDMs, in a dose-dependent manner (Figure 3A). Moreover, loss of MAVS reduced CXCL1 secretion from γHV68-infected BMDMs, albeit with boarder-line statistical significance (Figure 3A). Thus, upon γHV68 infection, MAVS deficiency does not elevate the intrinsic ability of BMDMs to produce cytokines.

Having excluded that MAVS deficiency elevates the intrinsic cytokine production by macrophages, we surmised that the elevated cytokines in γHV68-infected *Mavs* ^−/−^ mice, at least during early infection (e.g., 7 d.p.i.), are likely produced by lung epithelium/fibroblasts. Next, we tested whether MEFs recapitulate the MAVS-dependent avoidance of cytokine production and examined cytokines secreted from γHV68-infected MEFs by ELISA. γHV68 infection induced significantly more IL6 and CCL5 in *Mavs* ^−/−^ MEFs than *Mavs* ^+/+^ MEFs (Figure 3B), recapitulating the phenotypic cytokine production observed in γHV68-infected mice (Figure 1A). It is important to note that MAVS deficiency does not elevate cytokine production in mock-infected MEFs (Figure 3C). As expected, SeV infection induced much more IL6 in *Mavs* ^+/+^ MEFs than in *Mavs* ^−/−^ MEFs, confirming the MAVS-dependent cytokine production in response to infection by a prototype RNA virus (Figure 3D). Interestingly, we found that mRNA and protein levels of cytokines were inversely correlated with the levels of γHV68 replication in the lung of BL/6 mice (Figure S6), suggesting that γHV68 inhibits cytokine production at the transcription level.

### MAVS is Necessary for γHV68 Infection to Abrogate NFκB Activation

**Figure 4:**
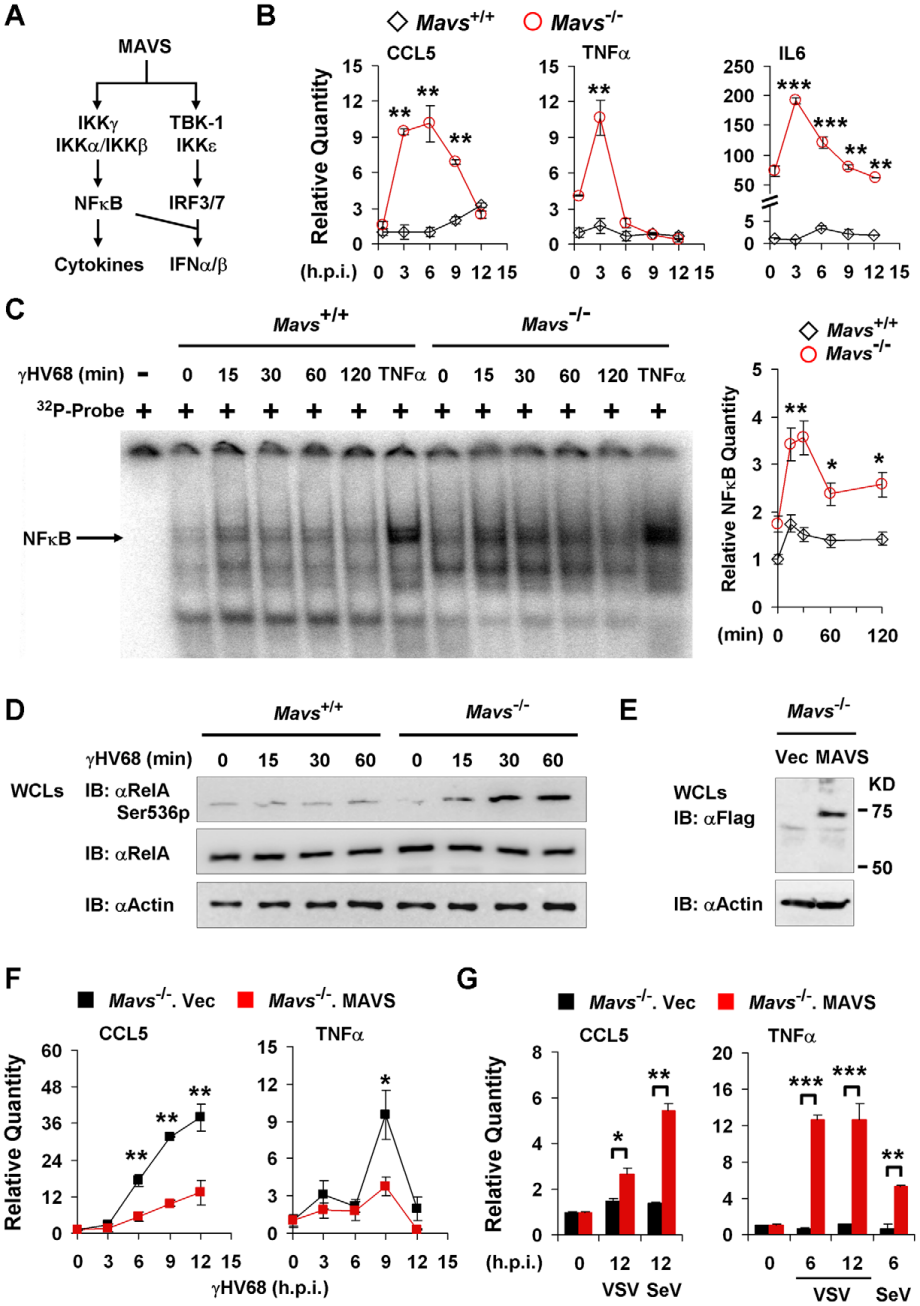
γHV68 abrogates NFκB activation in a MAVS-dependent manner. *Mavs* ^+/+^ and *Mavs* ^−/−^ mouse embryonic fibroblasts (MEFs) were infected with γHV68 (MOI = 5) (B, C, D, and F), vesicular stomatitis virus (VSV, MOI = 10) (G), or Sendai virus (SeV, 1000 HA units) (G). See also Figure S6. (A) Two main signaling pathways downstream of the MAVS adaptor. (B) Relative quantity of cytokine mRNAs in γHV68-infected MEFs. Cytokine mRNA levels were analyzed by real-time PCR and normalized to that of β-actin. See also Figure S7 and Figure S8. (C) Nuclear fractions (2 µg) were prepared from γHV68-infected or TNFα-treated MEFs and analyzed by electrophoresis mobility shift assay (EMSA) (*left*). The NFκB bands (*black arrow*) were quantified by densitometry and normalized to that of the mock-infected *Mavs* ^+/+^ MEFs (*right*). See also Figure S9. (D) Whole cell lysates (WCLs) of γHV68-infected *Mavs* ^+/+^ and *Mavs* ^−/−^ MEFs were analyzed by immunoblot with antibodies specific for the Ser536 phosphorylated (Ser536p) RelA, total RelA, and β-actin. (E to G) MAVS expression was “reconstituted” by lentivirus as described in Materials and Methods. (E) MAVS expression was confirmed by immunoblot with anti-Flag antibody. (F and G) Relative quantity of cytokine mRNAs in control (*Mavs* ^−/−^.Vec) or MAVS-reconstituted (*Mavs* ^−/−^.MAVS) MEFs infected with γHV68 (F), VSV (G) or SeV (G) were analyzed by real-time PCR as in (B). Data are presented as the mean ± SEM of three independent experiments. The statistical significance: *, *P*<0.05; **, *P*<0.02.

Two main signaling cascades, i.e., NFκB and IRF-IFN pathways, are known to relay MAVS-dependent innate immune activation (Figure 4A). In response to viral infection, NFκB activation downstream of MAVS and IKKβ is essential for gene expression and secretion of antiviral cytokines. Therefore, we assessed the mRNA levels of CCL5, IL6 and TNFα in γHV68-infected MEFs by quantitative real-time PCR. γHV68 infection robustly increased the mRNA abundance of all three cytokines within 6 hours post-infection (h.p.i.) in *Mavs* ^−/−^ MEFs, which was not observed in γHV68-infected *Mavs* ^+/+^ MEFs (Figure 4B). Moreover, loss of MAVS reduced IFN-β gene expression induced by γHV68 infection (Figure S7), indicating the specificity of MAVS utilization by γHV68. Finally, γHV68 infection failed to up-regulate gene expression of these inflammatory cytokines in MEFs deficient in IKKβ and IKKγ (Figure S8), consistent with the notion that activated IKKβ is necessary for cytokine production in response to viral infection. These results suggest that MAVS is necessary for γHV68 to prevent cytokine gene expression and that up-regulated gene expression likely underpins the increased cytokine secretion in *Mavs* ^−/−^ MEFs. Thus, we examined NFκB activation by electrophoresis mobility shift assay (EMSA). Agreeing with the elevated cytokine gene expression in *Mavs* ^−/−^ MEFs, γHV68 infection imparted more robust DNA-binding activity of NFκB in nuclear extract of *Mavs* ^−/−^ MEFs than that of *Mavs* ^+/+^ MEFs (Figure 4C). The specificity of EMSA for NFκB was confirmed by a competition assay using a cold probe and a super-shift assay using a RelA-specific antibody (Figure S9). Densitometry analysis further showed that the DNA-binding activity of NFκB, induced by γHV68 infection, in *Mavs* ^−/−^ MEFs was approximately two-fold of that in *Mavs* ^+/+^ MEFs (Figure 4C). RelA phosphorylation at serine 536 (Ser536) was demonstrated as a marker for NFκB activation [29]. We further examined the Ser536 phosphorylated RelA by immunoblot and found that γHV68 infection also triggered a robust accumulation of the Ser536 phosphorylated RelA in *Mavs* ^−/−^ MEFs, but not in *Mavs* ^+/+^MEFs (Figure 4D). To confirm that loss of MAVS is responsible for increased cytokine production induced by γHV68, we “reconstituted” MAVS expression by lentivirus in *Mavs* ^−/−^ MEFs (Figure 4E), and examined gene expression of cytokines (such as TNFα and CCL5) in response to viral infection. We found that the “reconstituted” expression of MAVS reduced cytokine gene expression triggered by γHV68 (Figure 4F), whereas up-regulated cytokine gene expression induced by VSV and SeV (Figure 4G), supporting the premise that loss of MAVS elevated γHV68-induced cytokine production. Collectively, these results indicate that MAVS is critical for γHV68 to negate NFκB activation and cytokine gene transcription in γHV68-infected cells.

### γHV68 Induces RelA Degradation in an MAVS- and IKKβ-dependent Manner

**Figure 5:**
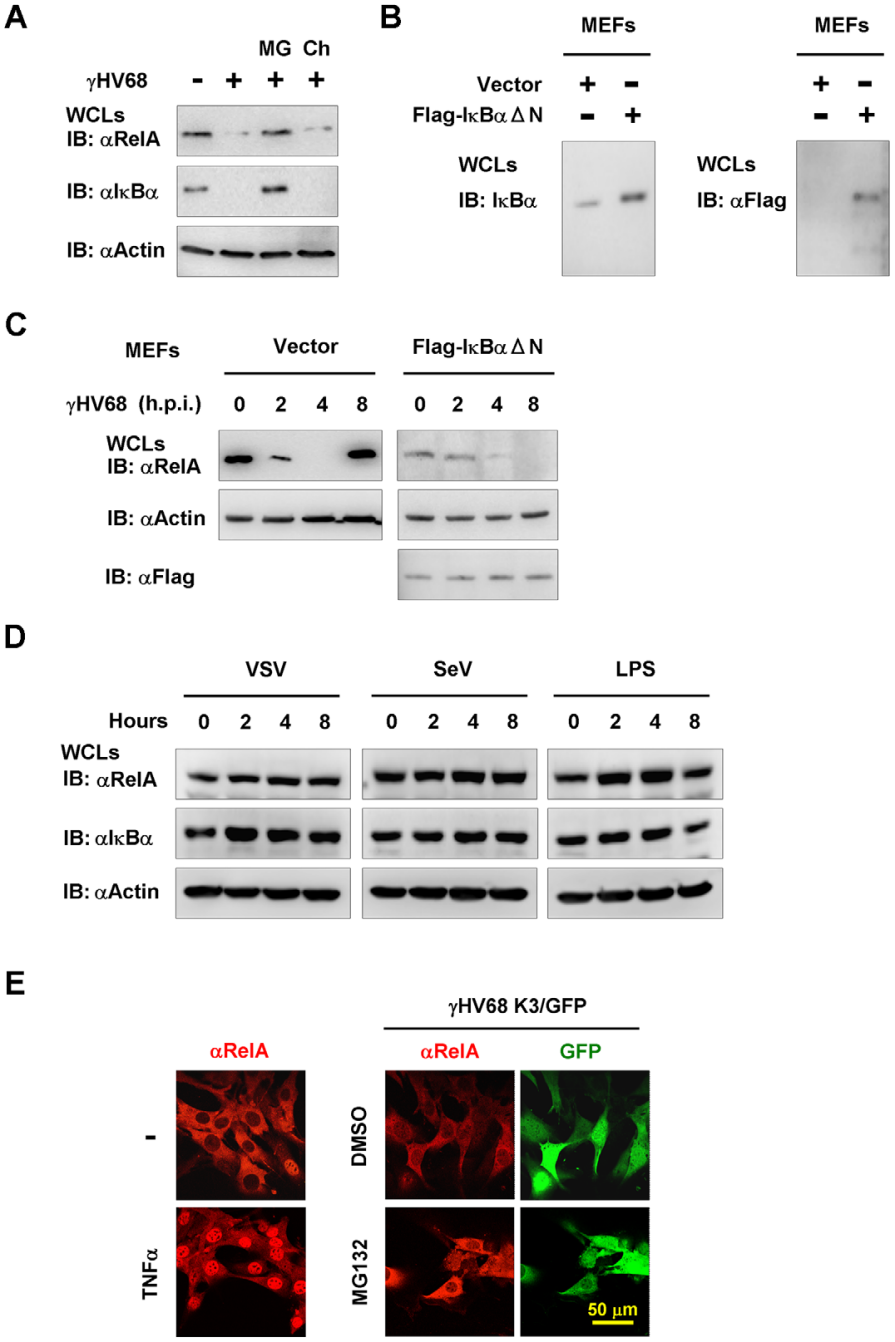
γHV68 infection induces RelA degradation in an IκBα-independent manner. Mouse embryonic fibroblasts (MEFs) were infected with γHV68 K3/GFP at an MOI of 20 (A, C, and E), or vesicular stomatitis virus (VSV, MOI = 10) (D), or Sendai virus (SeV, 4000 HA units) (D), or treated with lipopolysaccharides (LPS, 200 µg/ml). (A) Protein levels of RelA, IκBα, and β-actin in whole cell lysates (WCLs) from γHV68-infected wild-type MEFs at 4 h.p.i. were analyzed by immunoblot. MG, MG132 (20 µM); Ch, chloroquine (50 µM). (B and C) Wild-type MEFs stably expressing the Flag-tagged IκBα super-suppressor (IκBαΔN) were established as described in Materials and Methods. The expression of endogenous and exogenous IκBα was examined by immunoblot (B). WCLs of γHV68-infected control (Vector) or IκBαΔN-expressing MEFs were separately analyzed by immunoblot with indicated antibodies (C), to facilitate the detection of RelA that was reduced by IκBαΔN expression. Relative quantity of RelA in γHV68-infected MEFs was normalized to β-actin (*right*). See also Figure S10. (D) Wild-type MEFs were infected with VSV, SeV, or treated with LPS, and WCLs were analyzed by immunoblot with indicated antibodies. (E) MG132 treatment induced RelA accumulation in the cytosol of γHV68-infected MEFs. Wild-type MEFs were treated with TNFα (10 ng/ml, 30 min) (*left*) or infected with γHV68 without or with MG132 (20 mM) for 2 hours (*right*). A representative optical field is presented for each group. Data represent three independent experiments.

To dissect the molecular mechanism by which γHV68 uncouples NFκB activation from IKKβ activation, we examined RelA protein in γHV68-infected MEFs by immunoblot analysis. RelA is the most ubiquitously and abundantly expressed subunit of the transcriptionally active NFκB dimer. We found that γHV68 infection abolished RelA protein at 4 h.p.i. Moreover, treatment by the proteasome inhibitor MG132, but not by the lysosome inhibitor chloroquine, completely restored RelA protein (Figure 5A). These results indicate that γHV68 infection induces the proteasome-dependent degradation of RelA.

We noted that γHV68 infection also induced the degradation of the inhibitor of NFκB (IκBα), which serves as an indicator of IKKβ activation (Figure 5A). To test whether IκBα degradation is necessary for γHV68-induced RelA degradation, we established *Mavs* ^+/+^ MEFs stably expressing the IκBα super-suppressor, IκBαΔN, by lentivirus infection and confirmed IκBαΔN expression by immunoblot (Figure 5B). Notably, the IκBαΔN expression decreased the steady state level of RelA and we analyzed RelA protein in these two MEF cell lines separately. Surprisingly, in MEFs expressing the IκBαΔN, RelA protein gradually disappeared, albeit in a slower kinetics, with the progression of γHV68 infection (Figure 5C), indicating that IκBα degradation is dispensable for RelA degradation in γHV68-infected MEFs. As expected, IκBαΔN was not degraded in γHV68-infected MEFs, and IκBαΔN potently abrogated RelA nuclear translocation that was induced by TNFα treatment (Figure S10). RelA degradation was not observed after infection by VSV and SeV, nor after treatment with lipopolysaccharide (Figure 5D). Next, γHV68-induced RelA degradation was further examined by immunofluorescence microscopy after treatment with MG132. Consistent with the proteasome-mediated RelA degradation, MG132 treatment restored RelA protein in γHV68-infected MEFs, with significant accumulation in the cytosol (Figure 5E). Collectively, these findings support the conclusion that γHV68 induces RelA degradation in an IκBα-independent manner.

**Figure 6:**
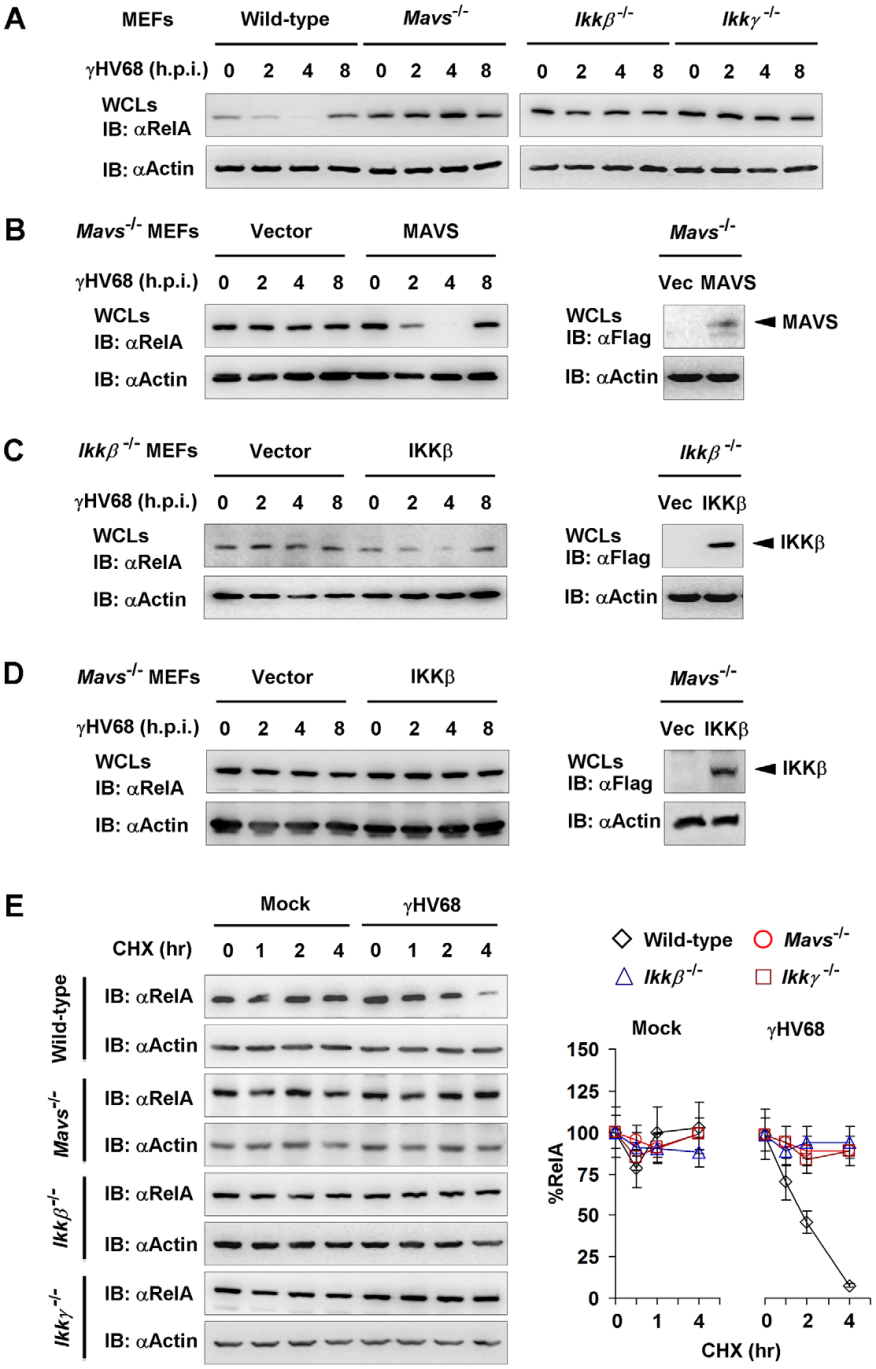
γHV68 infection induces transient RelA degradation in a MAVS- and IKKβ-dependent manner. Mouse embryonic fibroblasts (MEFs) of indicated genotypes were infected with γHV68 K3/GFP at an MOI of 20. Whole cell lysates (WCLs) were analyzed by immunoblot. (A) γHV68-infected MEFs were collected at various time points. RelA levels in WCLs were analyzed by immunoblot. See also Figure S11. (B and C) MAVS and IKKβ expression was “reconstituted” by lentivirus as described in Materials and Methods. MAVS or IKKβ expression was confirmed by immunoblot (*right*). Viral infection and immunoblot analyses for RelA proteins were carried out as in (A). (D) Exogenous IKKβ was expressed by lentivirus. WCLs of *Mavs* ^−*/*−^.Vec or *Mavs* ^−*/*−^.IKKβ infected with γHV68 were analyzed by immunoblot with antibodies to RelA and β-actin. (E) MEFs were mock-infected or infected with γHV68 for 30 min, and treated with cycloheximide (CHX, 20 µg/ml). WCLs were analyzed by immunoblot for RelA and β-actin. Relative quantity of RelA in γHV68-infected MEFs was normalized to β-actin (*right*). Data represent three independent experiments.

NFκB activation and cytokine gene expression induced by γHV68 appear to be transient in *Mavs* ^−/−^ MEFs, implying the dynamic regulation of the NFκB pathway by γHV68 infection. We assessed the kinetics of RelA protein in wild-type, *Mavs* ^−/−^, *Ikkβ* ^−/−^, and *Ikkγ* ^−/−^ MEFs infected with γHV68. Within 4 h.p.i., RelA protein gradually diminished in wild-type MEFs (Figure 6A). Remarkably, RelA protein re-appeared at 8 h.p.i. in wild-type MEFs, suggesting a transient RelA degradation triggered by γHV68 infection. However, relatively equivalent RelA protein was maintained in *Mavs* ^−/−^, *Ikkβ* ^−/−^, and *Ikkγ* ^−/−^ MEFs with the progression of γHV68 infection (Figure 6A). It is noteworthy that γHV68-induced RelA degradation in *Ikkα* ^−/−^ MEFs was comparable to that in *Mavs^+/+^* MEFs (Figure S11), implying that IKKα is dispensable for RelA degradation induced by γHV68. These results imply that the integral MAVS-IKKβ signaling node is important for γHV68 to induce transient RelA degradation. When exogenous MAVS and IKKβ were re-introduced by lentivirus infection, γHV68 infection induced a transient RelA degradation (Figures 6B,6C), bolstering the specific requirement for MAVS and IKKβ in RelA degradation triggered by γHV68 infection. To determine whether elevated IKKβ is sufficient for RelA degradation induced by γHV68 infection, we expressed exogenous IKKβ with lentivirus in *Mavs* ^−/−^ MEFs and found that γHV68 infection failed to reduce RelA protein (Figure 6D). Thus, this result supports the conclusion that the MAVS-dependent IKKβ activation, rather than the absolute IKKβ level, is necessary for γHV68-induced RelA degradation.

To further characterize the γHV68-induced RelA degradation, we determined the half-life of RelA in γHV68-infected MEFs in the presence of cyclohexamide (CHX), an inhibitor of protein translation. For this experiment, MEFs were infected with γHV68 for 30 minutes and CHX was added to halt protein synthesis. In γHV68-infected *Mavs* ^+/+^ MEFs, the half-life of RelA was reduced to approximately 2 hours (Figure 6E). CHX treatment, up to 4 hours, did not significantly alter RelA protein level in mock-infected MEFs. Furthermore, RelA protein remained relatively constant in γHV68-infected MEFs deficient in MAVS, IKKβ, or IKKγ (Figure 6E). Taken together, these results indicate that MAVS and IKKβ are necessary for γHV68 to induce rapid RelA degradation. Based on these findings, we conclude that γHV68 induces RelA degradation in a MAVS- and IKKβ-dependent, and IκBα-independent manner.

### γHV68 Usurps MAVS and IKKβ to Induce the Site-specific (Serine 468) Phosphorylation of RelA

**Figure 7:**
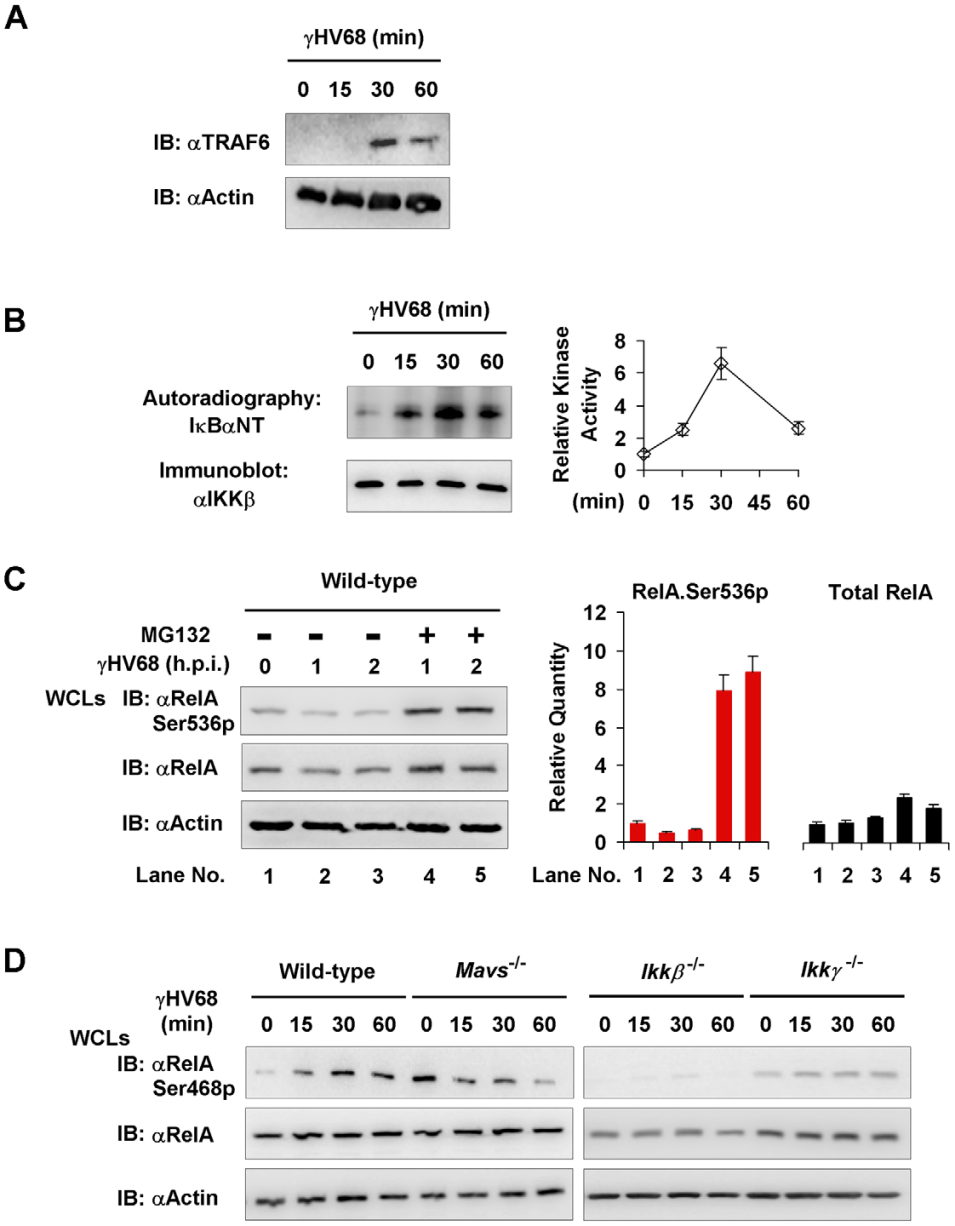
MAVS and IKKβ are important for γHV68-induced RelA phosphorylation at serine 468. Mouse embryonic fibroblasts (MEFs) of indicated genotypes were infected with γHV68 K3/GFP at an MOI of 20. (A) *Mavs* ^+/+^ MEFs were harvested at indicated time points and 20 mg of Triton X-100-insoluble fractions were analyzed by immunoblot with antibodies to TRAF6 and β-actin. (B) IKKβ was precipitated from γHV68-infected *Mavs* ^+/+^ MEFs for an *in vitro* kinase assay. Phosphorylation of the IκBα N-terminus (IκBαNT) was analyzed by autoradiography. Relative intensity of phosphorylated IκBαNT was normalized to IKKβ protein (*right*). (C) *Mavs* ^+/+^ MEFs were infected with γHV68 for 30 min, treated with MG132 (20 µM) for indicated time. Whole cell lysates (WCLs) were analyzed by immunoblot with indicated antibodies. Total RelA and Ser536 phosphorylated RelA (RelA.Ser536p) were determined by densitometry analysis and normalized to β-actin (right). (D) WCLs of γHV68-infected MEFs were analyzed by immunoblot with antibodies specific for Ser468 phosphorylated (Ser468p) RelA, or total RelA, and β-actin. Data represent three independent experiments.

The MAVS- and IKKβ-dependent, IκBα-independent RelA degradation triggered by γHV68 infection prompted us to hypothesize that IKKα directly phosphorylates RelA to facilitate its turnover. Although IKKβ is historically known for IκBα phosphorylation and subsequent degradation, recent studies also implicated IKKβ and IKKα in phosphorylating RelA and terminating NFκB activation. Two predominant IKKβ-mediated phosphorylation sites, i.e., Serine 536 (Ser536) and Serine 468 (Ser468), have been implicated in regulating RelA turnover [29], [30], [31], [32], [33]. Because we observed significant reduction of RelA protein levels at 2 h.p.i. (Figures 6A), we reasoned that RelA phosphorylation by IKKβ precedes its degradation and focused on the first hour post γHV68 infection. To further characterize the activation of the IKKβ-NFκB ramification downstream of MAVS, we have examined TRAF6 translocation and kinase activity of IKKβ after γHV68 infection. We first determined the migration of TRAF6 into the Triton X-100-insoluable fraction that marks TRAF6 activation. Indeed, TRAF6 was detected at 30 and 60 minutes post-infection in the Triton X-100 insoluble pellet of γHV68-infected MEFs, but not in that of mock-infected MEFs (Figure 6A). We further assessed IKKβ activation by γHV68 infection with an *in vitro* kinase assay. Strikingly, γHV68 infection potently up-regulated IKKβ kinase activity, as demonstrated to phosphorylate the IkBa N-terminal sequence *in vitro*, as early as 15 min post-infection (Figure 7B). These results indicate that γHV68 infection sufficiently instigates the activation of the MAVS-IKKβ pathway.

Next, we examined the RelA phosphorylation at two IKKβ phosphorylation sites, i.e., Ser468 and Ser536, in γHV68-infected MEFs. We found an accumulation of the Ser536 phosphorylated RelA in γHV68-infected *Mavs* ^−/−^ MEFs, but not in *Mavs* ^+/+^ MEFs (Figure 4D), which was consistent with more robust NFκB activation in *Mavs* ^−/−^ MEFs than those in *Mavs* ^+/+^ MEFs (Figure 4C). These results suggest that Ser536-phosphorylated (Ser536p) RelA represents the activated RelA, and is selectively being targeted for degradation. We therefore infected MEFs with γHV68 and treated with MG132 to inhibit protein degradation. After treatment for 1 hour, the Ser536 phosphorylated RelA was increased by more than 8-fold, whereas total RelA was only increased approximately 2-fold (Figure 7C). This result indicates that activated RelA is being selectively degraded by γHV68 infection. Consistent with the pivotal role of the Ser468 phosphorylated form in promoting RelA degradation, we found that the Ser468p RelA gradually increased in *Mavs* ^+/+^ MEFs, and conversely decreased in *Mavs* ^−/−^ MEFs, infected with γHV68 (Figure 7D). The Ser468p RelA, under both basal and γHV68-infected conditions, was not observed in *Ikkβ* ^−/−^ MEFs and severely impaired in *Ikkγ* ^−/−^ MEFs (Figure 7D), indicating that activated IKKβ is necessary for RelA phosphorylation at Ser468. The distinct pattern of RelA phosphorylation in γHV68-infected MEFs, dependent on MAVS expression (Figures 4D,7D), suggests the site-specificity of RelA phosphorylation instigated by γHV68. These results also agree with the requirement of IKKβ activation for RelA degradation triggered by γHV68 (Figure 6), linking RelA Ser468 phosphorylation to its degradation.

### The MAVS-dependent RelA Phosphorylation at Ser468 is Necessary for γHV68-induced RelA Degradation

**Figure 8:**
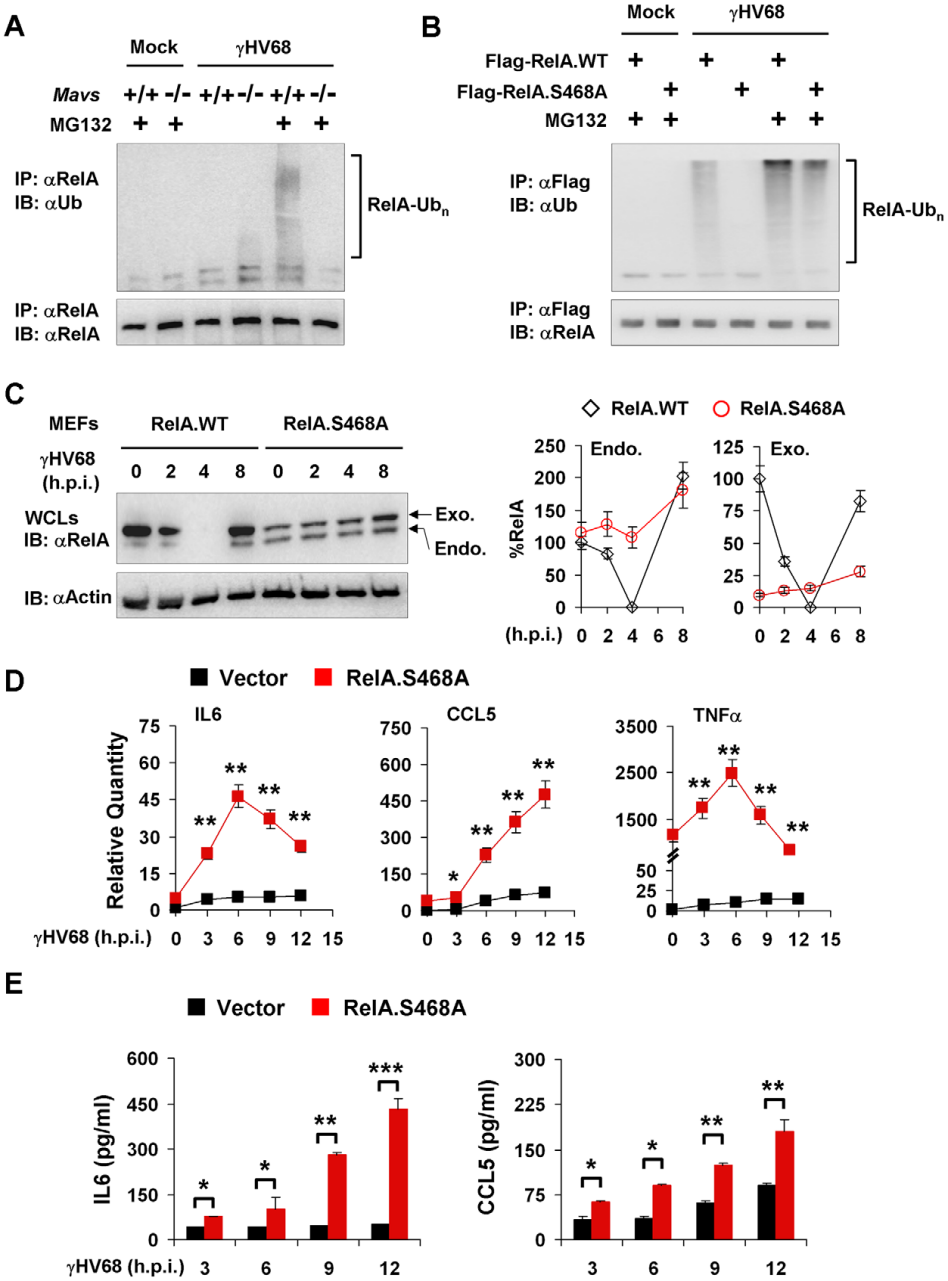
RelA phosphorylation at Serine 468 is critical for γHV68-induced RelA degradation. Mouse embryonic fibroblasts (MEFs) were infected with γHV68 K3/GFP at an MOI of 20 (A, B, and C) or 5 (D and E). (A) At 2 hours post-infection, mock- or γHV68-infected *Mavs* ^+/+^ and *Mavs* ^−/−^ MEFs were treated with MG132 (20 µM) for an hour or left un-treated. Endogenous RelA was precipitated and analyzed by immunoblot with indicated antibodies. (B) *Mavs* ^+/+^ MEFs stably expressing Flag-tagged wild-type RelA (RelA.WT) or the RelA.S468A variant were established as described in Materials and Methods. MEFs were infected with γHV68 and treated with MG132 (20 µM) as in (A). Exogenous RelA was precipitated with anti-Flag antibody and analyzed by immunoblot with indicated antibodies. (C) Whole cell lysates (WCLs) from γHV68-infected *Mavs* ^+/+^ MEFs stably expressing wild-type RelA or the RelA.S468A variant were analyzed by immunoblot. Endo., endogenous RelA; Exo., exogenous RelA. Relative quantity of RelA was normalized to β-actin. Percentage of RelA protein was calculated in reference to that of uninfected MEFs (RelA) (*right*). (D and E) Control (Vector) or RelA.S468A-expressing *Mavs* ^+/+^ MEFs were infected with γHV68 for indicated time. (D) Cytokine mRNA levels were analyzed by real-time PCR and normalized to that of β-actin. (E) Cytokine levels in the supernatant were determined by ELISA. Data are presented as the mean ± SEM of three independent experiments. The statistical significance: *, *P*<0.05; **, *P*<0.02; ***, *P*<0.005. See also Figure S12.

RelA phosphorylation at Ser468 represents a key step in promoting RelA ubiquitination, we then assessed the ubiquitination of RelA by immuno-precipitation with anti-RelA antibody and immunoblot with anti-ubiquitin antibody. When treated with MG132 for two hours, γHV68-infected *Mavs* ^+/+^ MEFs accumulated detectable levels of high molecular species shown by the smearing of RelA, indicative of RelA ubiquitination (Figure 8A). By contrast, the smeared RelA proteins were not observed in γHV68-infected *Mavs* ^−/−^ MEFs (Figure 8A). In the absence of MG132, ubiquitinated RelA was not detected in both *Mavs* ^+/+^ and *Mavs* ^−/−^ MEFs with or without γHV68 infection. The fact that RelA ubiquitination was only detected with MG132 treatment in γHV68-infected *Mavs* ^+/+^ MEFs implies that RelA ubiquitination is the rate-limiting step in the process of degrading RelA.

To determine whether the Ser468 phosphorylation of RelA is necessary for its degradation, we established *Mavs* ^+/+^ MEFs that stably express the RelA variant carrying the Serine 468-to-Alanine mutation (RelA.S468A) or wild-type RelA by lentivirus infection, and assessed RelA ubiquitination in γHV68-infected MEFs. We found that the exogenously expressed wild-type RelA was efficiently ubiquitinated, and that the S468A mutation abrogated the ubiquitination of the RelA.S468A variant in γHV68-infected *Mavs* ^+/+^ MEFs, supporting the proposition that the Ser468 phosphorylation is necessary for efficient RelA ubiquitination (Figure 8B). When γHV68-infected *Mavs* ^+/+^ MEFs were treated with MG132, the ubiquitinated RelA.S468A was observed at a lower level than the ubiquitinated wild-type RelA (Figure 8B). Next, the effect of the RelA.S468A variant on γHV68-induced RelA degradation was assessed. The exogenous RelA.S468A variant, although expressed at a lower level than wild-type RelA, was resistant to degradation induced by γHV68 infection, but wild-type RelA was not (Figure 8C). Interestingly, the exogenously expressed RelA.S468A variant also protected endogenous RelA from γHV68-induced degradation (Figure 8C), suggesting that the RelA.S468A variant functions as a dominant negative of RelA degradation.

To evaluate the contribution of transient RelA degradation to the reduced cytokine production, we determined whether the expression of the RelA.S468A variant, which inhibited RelA degradation, is sufficient to up-regulate cytokine gene expression in response to γHV68 infection. We used quantitative real-time PCR analysis to examine the mRNA levels of IL6, CCL5 and TNFα. We have noticed that the expression of the RelA.S468A variant had a marginal effect on the basal level of mRNAs of IL6 and CCL5, whereas the RelA.S468A expression elevated basal TNFα mRNA over 1,000 fold (Figure 8D). Regardless of the basal mRNA levels, the expression of the RelA.S468A variant greatly increased IL6 and CCL5 mRNA levels induced by γHV68 infection (Figure 8D). Although the basal TNFα mRNA was exceedingly high in MEFs expressing the RelA.S468A variant, γHV68 infection further increased TNFα mRNA to approximately 2,500 fold (Figure 8D). Consistent with the up-regulation of cytokine gene expression, the RelA.S468A variant also increased IL6 and CCL5 secretion in γHV68-infected MEFs (Figure 8E). Notably, despite that TNFα mRNA was highly up-regulated by RelA.S468A and γHV68 infection, TNFα secretion was under detection in MEFs (data not shown). Finally, the exogenous expression of the RelA.S468A variant reduced γHV68 lytic replication under both permissive and restricted (methylcellulose-containing) conditions (Figure S12). These results suggest that inhibiting RelA degradation is sufficient to restore cytokine gene expression and production in γHV68-infected *Mavs* ^+/+^ MEFs, and demonstrate a requisite role of the Ser468 phosphorylation in efficient RelA ubiquitination and degradation induced by γHV68. Our findings uncover an essential role of MAVS in specifying the site-specific (Ser468) phosphorylation of RelA to promote RelA degradation and terminate NFκB activation, thereby effectively preventing cytokine production induced by γHV68 infection.

## Discussion

Recent studies have demonstrated that, in response to viral infection, the MAVS adaptor protein relays innate immune signaling from cytosolic sensors to NFκB and IRF activation that up-regulate antiviral cytokine production. Mice deficient in MAVS are severely compromised in host defense against infection of several viruses [23], [24], [34]. Moreover, viruses of the positive-stranded RNA family target MAVS for destruction to disable host innate immune responses [9], [35], [36], [37], [38]. In this study, we report that γHV68 hijacks MAVS and its immediately downstream IKKβ kinase to degrade RelA, a key subunit of the transcriptionally active NFκB dimer. In doing so, γHV68 effectively terminates NFκB activation and negates cytokine production. To our knowledge, this is the first example whereby signaling via the upstream components of the innate immune pathways, MAVS and IKKβ, is intercepted by a pathogen to degrade the critical downstream effector, RelA, a master transcription factor that governs the expression of a myriad of genes of immune function. Given the common biological properties shared by members of the gamma herpesvirus family, it is possible that human KSHV and EBV have evolved equivalent tactics to evade cytokine production.

### An Intricate Immune Evasion Strategy: Hijacking Innate Immune Signaling Activation to Abrogate Cytokine Production

Our observation that the MAVS-IKKβ pathway, which otherwise activates NFκB and promotes cytokine production, is directed by γHV68 to assist in degrading RelA and terminating NFκB activation is surprising. Moreover, γHV68 infection resulted in elevated cytokines in mice and fibroblasts that are deficient in MAVS, indicating that MAVS is an integral player of the active evasion scheme to abrogate host cytokine production. The elevated cytokine production in MAVS-deficient mice and MEFs, in response to γHV68 infection, is opposite to what was observed for RNA virus infection [23], [24]. Our study thus highlights an unrecognized role of MAVS and IKKβ in terminating NFκB activation. These findings also explain an early report that IL6 deficiency had no apparent effect on γHV68 infection [26], in that γHV68 effectively negates cytokine (such as IL6) production during early viral infection. Presumably, the ability of γHV68 to abolish cytokine production contributes to the minimal, if any, effect of IL6 deficiency on γHV68 lytic replication, and implies that physiological function of cytokines against γHV68 may be better defined in mouse strains (e.g., *Mavs* ^−/−^ mouse) that γHV68 infection induces more antiviral cytokines. Alternatively, exogenous cytokines may be delivered to γHV68-infected mice during early infection when cytokines are not produced. Indeed, we showed that treatment with IL6 and TNFα greatly reduced γHV68 replication *in vivo* and *ex vivo*, demonstrating their antiviral activity against γHV68 lytic replication.

The significance of elevated inflammatory cytokines, which were approximately two-fold of those in *Mavs* ^+/+^ mice, is substantiated by escalated immune cell infiltration in the lung of *Mavs* ^−/−^ mice. Evidently, γHV68 infection resulted in significantly more infiltrated immune cells in the lung of *Mavs* ^−/−^ mice than those of *Mavs* ^+/+^ mice. It is important to point out that West Nile virus was recently reported to induce an uncontrolled inflammatory response in *Mavs* ^−/−^ mice, including a signature of higher serum levels of multiple inflammatory cytokines [34]. The elevated cytokine production likely stems from an increased viral load, and is further compounded by the lack of negative feedback mechanisms on host immune responses. By contrast, γHV68 replicated to relatively equivalent (7 d.p.i.) or lower (10 d.p.i.) viral loads in *Mavs* ^−/−^ mice than in *Mavs* ^+/+^ mice. This observation excludes the contribution of higher viral loads in *Mavs* ^−/−^ mice to the elevated cytokine production and further emphasizes the roles of MAVS in evading cytokine production by γHV68. Conceivably, loss of MAVS in lung fibroblasts/endothelial cells, which support γHV68 lytic replication, increases the cytokine production during early γHV68 infection (e.g., 7 d.p.i.). One notable common property of lung fibroblasts and embryonic fibroblasts is the abundant expression of the innate immune signaling components, providing a physiological rationale to dissect signal transduction in γHV68-infected MEFs. In fact, we recapitulated the MAVS-dependent reduction in cytokine secretion using γHV68-infected MEFs. By contrast, bone marrow-derived macrophages of *Mavs* ^−/−^mice secreted either similar or lower levels of cytokines than those of *Mavs* ^+/+^ mice in response to γHV68 infection. Based on these observations, we propose that γHV68-induced cytokines in the lung are produced in a biphasic process: the initial low-level production by lung fibroblasts and more robust production by infiltrated immune cells during late stages of γHV68 infection. Within this scenario, cytokines of the initial phase recruit and stimulate the proliferation of immune cells (e.g., macrophages and neutrophils) that release more cytokines during late infection, such as at 13 and 16 d.p.i., when replicating γHV68 was cleared. These results collectively indicate that loss of MAVS elevates host innate immune responses against γHV68 and support the conclusion that elevated immune responses, in turn, negatively impact the lytic replication of γHV68 in *Mavs* ^−/−^ mice.

Manipulating the host immune response has been a recurring theme for host-pathogen interactions [39], [40]. As intracellular pathogens, viruses are obligate to utilize host components to achieve efficient replication and dissemination. Host innate immunity is the first line of defense that plays critical roles in containing invading viruses during early viral infection. We have witnessed a growing list of strategies by which pathogens deploy to evade host innate immune responses [41], [42], [43], [44]. Many of which are evolved to thwart cytokine production or signal transduction thereof, such as IFNs. Although negating NFκB activation by pathogens has been reported previously, our study showing that MAVS and IKKβ are hijacked to promote RelA phosphorylation and degradation unravels an active evasion strategy whereby activation of upstream signaling events are exploited to nullify the immediate downstream event. Given the fundamental functions of innate immune signaling pathways in cellular physiology, the discovery that pathogens exploit these signaling events for their own benefit is not a complete surprise and emerging studies support this evolving theme [45], [46], [47].

### An Essential Role of MAVS in NFκB Termination

One of the well-defined mechanisms that regulate NFκB activation is the phosphorylation and degradation of IκBs. For example, activated IKKβ phosphorylates IκBα to induce its degradation, unleashing NFκB that promotes the transcription of various genes, including IκBα. γHV68 infection appears to activate IKKβ that, in turn, triggers the degradation of both IκBα and RelA. Intriguingly, these two seemingly coupled processes are independent from each other and IκBα degradation is dispensable for RelA degradation in γHV68-infected cells. Evidently, γHV68 infection effectively uncouples NFκB activation from IKKβ activation by inducing RelA degradation in an IκBα-independent manner. Krug et al. previously reported that the expression of the IκBα super-suppressor had no apparent effect on γHV68 lytic replication [48], whereas RelA was found to inhibit γHV68 lytic replication [25]. Thus, the IκBα-independent degradation of RelA, triggered by γHV68, offers a plausible interpretation for the apparent paradoxical effect of RelA and the IκBα super-suppressor on γHV68 lytic replication. Finally, data from our studies employing the IκBαΔN super-suppressor and MG132 treatment imply that γHV68 induces RelA degradation in the cytosol, although further investigation is necessary to address this possibility. Altogether, these studies are en route to assemble an overall picture regarding the dynamic regulation of NFκB-dependent gene transcription in γHV68 lytic replication.

Besides IκBs, IKKβ differentially regulates NFκB activation *via* phosphorylation of two serine residues within the carboxyl terminal region of RelA, i.e., Ser468 and Ser536. Whereas the Ser536 phosphorylation of RelA potentiates NFκB-dependent gene transcription through recruiting p300 [49], the Ser468 phosphorylation instigates RelA degradation [31], [32], [33]. It is noteworthy that RelA phosphorylation at Ser536 by IKKα was reported to facilitate RelA degradation in macrophages [30]. In this study, we found that γHV68 infection induced a gradual increase of the Ser468p RelA in *Mavs* ^+/+^ MEFs, whereas a gradual decrease of the Ser468p RelA was observed in γHV68-infected *Mavs* ^−/−^ MEFs. Importantly, the phosphorylated forms of RelA, e.g., Ser468p and Ser536p, account only a minor fraction of the total pool of RelA and that the bulky part of RelA remains constant. In fact, RelA depletion by γHV68 infection requires a high MOI (MOI = 20), although NFκB termination necessitates a relatively lower MOI (MOI = 5). The increase of the Ser468p RelA correlated with more efficient ubiquitination and rapid degradation of RelA in γHV68-infected *Mavs* ^+/+^ MEFs. Conversely, the increase of the Ser536p RelA correlated with NFκB activation and the up-regulated gene expression of inflammatory cytokines in γHV68-infected *Mavs* ^−/−^ MEFs. The key roles of the S468 phosphorylation of RelA is further substantiated by the observations that the expression of the RelA.S468A variant sufficiently inhibited γHV68-induced RelA degradation and restored cytokine gene expression. Collectively, these results support the corollary that γHV68 usurps MAVS to facilitate RelA phosphorylation at Ser468, which primes RelA for the proteasome-mediated degradation in *Mavs* ^+/+^ MEFs. We observed that MG132 treatment greatly increased the Ser536p RelA, but exhibited much less effect on total RelA protein, favoring the possibility that the Ser536p RelA may be specifically targeted for Ser468 phosphorylation in γHV68-infected *Mavs* ^+/+^ MEFs. Nevertheless, our findings highlight a critical role of MAVS in the site-specific phosphorylation (Ser468) of RelA to promote RelA degradation and terminate NFκB-dependent gene transcription.

In response to γHV68 infection, the NFκB activation and cytokine gene expression in *Mavs* ^−/−^ MEFs is transient, suggesting that γHV68 may exploit additional unknown signaling pathways to evade NFκB-mediated cytokine production. One candidate is the MyD88-dependent pathway, which is supported by a recent study showing that KSHV utilizes TLR7 to promote viral reactivation from latency. Mouse and MEFs deficient in MAVS and/or MyD88 will enable the interrogation of these two key adaptor molecules in γHV68 infection. A closely-related and logical extension of the above question is how γHV68 is sensed to promote NFκB activation and cytokine production in MAVS-deficient mouse and MEFs. Although MAVS is dispensable, IKKβ is absolutely required for γHV68-induced NFκB activation and cytokine production, consistent with the notion that IKKβ is a major antiviral innate immune kinase responsible for NFκB activation. Additional experiments are under way to identify potential pathways that induce NFκB activation in a MAVS-independent manner.

### Viral Manipulation of the NFκB Pathway

Although IKKα and IKKβ are primarily responsible for NFκB activation downstream of a broad spectrum of physiological stimuli, IKK kinases are equally essential for NFκB termination. Through the exact same kinases, the opposing outcomes of NFκB transcription factors are likely engendered by distinct upstream signaling cascades. Therefore, biochemical studies characterizing these signaling events that differentially provoke either NFκB activation or NFκB termination will reveal insight into the mechanisms governing NFκB-mediated transcription. Such mechanisms are expected to dictate the amplitude and duration of inflammatory immune responses. It is very likely that host-virus interactions upstream of MAVS, when coupled with a downstream viral effector(s), efficiently induce RelA degradation. Several E3 ligases have been reported to promote RelA degradation under various conditions [32], [50], [51], [52], however, the cellular E3 ligases that are responsible for IKKα-dependent RelA degradation in macrophage remain unknown. Regardless of the nature of the cellular E3 ligases involved in γHV68-induced RelA degradation, it will be informative to identify the viral factor(s) that couples RelA degradation to the activation of the MAVS-IKKβ pathway during early γHV68 infection. The fact that cyclohexamide treatment, at 30 min post-infection, did not abolish γHV68-induced RelA degradation implies that a structural component(s) of the incoming virion is capable of accelerating RelA turnover. However, we can not exclude the immediately early viral gene products that are likely expressed within 30 min post-infection when cells are infected at an MOI of 20. Recently, the latent nuclear antigen LANA of γHV68 and RTA of KSHV were reported to degrade RelA and IRF3/7, respectively [53], [54]. Experiments are under way to identify viral factors (including LANA and RTA) that induce RelA degradation. Together, information obtained from these studies will provide an overview on the dynamic and intricate regulation of NFκB activation in γHV68 infection, guiding us to design better studies on human KSHV and EBV.

It is important to point out that the innate immune activation and NFκB manipulation by γHV68 occur immediately after viral infection, temporal period that we know very little. Conceivably, the effect of cytokines and the fate of incoming virions are determined within the very early phase immediately after viral infection. Indeed, γHV68 potently activated IKKβ within 15 to 30 minutes post-infection, which correlated with the Ser468 phosphorylation of RelA. Presumably, the activation kinetics of the MAVS-IKKβ pathway depends on the multiplicity of infection per cell, and higher doses of infectious γHV68 favor earlier and more robust activation of IKKβ and termination of NFκB.

**Figure 9:**
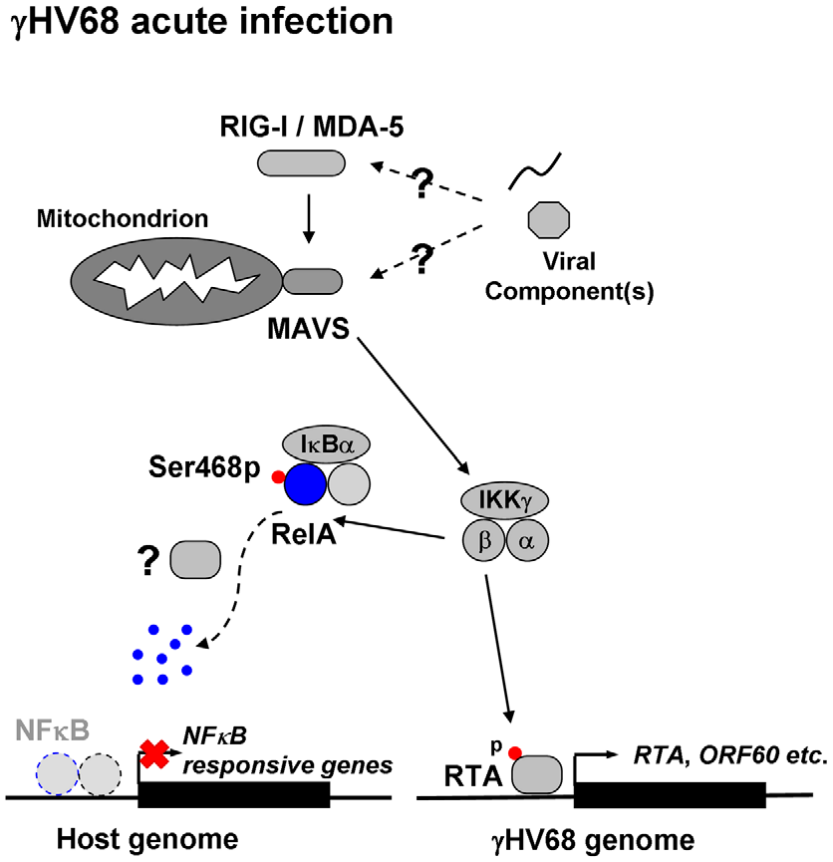
Dual roles of MAVS and IKKβ in γHV68 lytic replication. γHV68 infection activates MAVS and IKKβ. Activated IKKβ phosphorylates viral RTA to promote transcriptional activation (*right*), and cellular RelA to abrogate NFκB activation (*left*). In doing so, γHV68 infection effectively couples viral transcriptional activation to innate immune activation, whereas it uncouples NFκB activation from immediately upstream innate signal transduction to negate antiviral cytokine production. The sum of both culminates in enhancing γHV68 lytic replication. Question marks indicate unknown viral factors involved in innate immune activation and RelA degradation.

We have previously demonstrated that γHV68 hijacks the MAVS-IKKβ pathway to promote viral transcriptional activation *via* RTA phosphorylation by IKKβ [22]. We report here that γHV68 exploits MAVS and IKKβ to promote RelA degradation and NFκB termination, thereby preventing antiviral cytokine production. Collectively, our findings argue for the corollary that γHV68 has evolved a “one stone, two birds” strategy to harness host innate immune activation through coupling viral transcription activation and cellular NFκB termination to the MAVS-IKKβ pathway, thereby enabling viral lytic replication while disabling host cytokine production (Figure 9). It is possible that RelA degradation and RTA-dependent transcriptional activation are inherently coupled to promote γHV68 lytic replication. As such, γHV68 replicates more efficiently in *Mavs* ^+/+^ mice than in *Mavs* ^−/−^mice. However, it should be noted that MAVS is critical for antiviral cytokine production as demonstrated by previous studies using RNA viruses and this study with isolated macrophages. Thus, the phenotypes of γHV68 infection *in vivo* and *ex vivo* likely represent “neutralized” outcome of the anti- and pro-viral activities of MAVS in γHV68 infection. Regardless, our studies uncover intricate viral exploitation mechanisms of host innate immune signal transduction.

## Materials and Methods

### Ethics Statement

All animal work was performed under strict accordance with the recommendations in the Guide for the Care and Use of Laboratory Animals of the National Institutes of Health. The protocol was approved by the Institutional Animal Care and Use Committee (IACUC) of the University of Texas Southwestern Medical Center (permit number: A3472-01).

### Cell Lines and Viruses

NIH 3T3 cells, 293T cells, and mouse embryonic fibroblasts (MEFs) were maintained in DMEM (Mediatech) containing 8% newborn calf serum (NCS) or fetal bovine serum (FBS), respectively. Wild-type, *Mavs* ^−/−^, *Ikkα* ^−/−^, *Ikkβ* ^−/−^, and *Ikkγ* ^−/−^ MEFs were described previously [22], [23]. Bone marrow-derived macrophages (BMDMs) were obtained from *Mavs* ^+/+^ and *Mavs* ^−/−^ littermate mice, and cultured in DMEM containing 10% FBS and 10% L929 cell-conditioned medium for 6 days before viral infection. γHV68 K3/GFP was kindly provided by Dr. Philip Stevenson (Cambridge University, UK). Wild-type γHV68 and γHV68 K3/GFP were amplified in NIH 3T3 cells, and viral titer was determined by a plaque assay using NIH 3T3 monolayer. Sendai virus (SeV) stock (Charles River Laboratories) is 4000 HA units/ml.

### Mice and Infections

Wild-type (*Mavs* ^+/+^) and knockout (*Mavs* ^−/−^) mice were described previously [23]. Gender-matched, 6- to 8-week-old littermate mice were intranasally inoculated with 40 plaque-forming units (PFU) of wild-type γHV68. To assess cytokine production in the lung, gender- and age-matched BL/6 mice (ARC, UT Southwestern Medical Center) were intranasally infected with 1×10^5^ PFU of γHV68. To assess the antiviral effect of IL6 and TNFα, BL/6 mice were intranasally infected with 40 PFU of γHV68, and recombinant mouse IL6 and TNFα (rmIL6 and rmTNFα, PeproTech) were intranasally administered in 30 µl of 1% BSA (Sigma) in PBS from 1 to 5 days post-infection (d.p.i.) (30 ng/mouse/day). Mouse lungs were harvested at 6 d.p.i. and homogenized in DMEM.

### Determining the Delivery Efficiency of Intranasal Administration

To determine the delivery efficiency of intranasal administration of cytokines, 1×10^9^ relative light units (RLU) of firefly luciferase was diluted in 15 µl or 30 µl buffer (1% BSA in PBS). BL/6 mice were anaesthetized by intraperitoneally injecting a cocktail of ketamine and xylazine. Buffer alone or luciferase solution was intranasally administrated. Mouse tissues (nasal cavity, trachea, and lung) were harvested at 2 hours post administration, and homogenized by bead-beating in 300 µl passive lysis buffer (Promega). Luciferase activity was immediately quantified with the Luciferase Assay System (Promega). The delivery efficiency of intranasal administration was assessed by analyzing the relative luciferase activity in the lung.

### Cytokine ELISA

Commercial cytokine ELISA kits used in this study include: IL6 (BD Bioscience), IL10 (BD Bioscience), TNFα (BD Bioscience), CCL5 (PeproTech), and CXCL1 (R&D Systems). Cytokine levels in mouse tissue samples or the supernatant from cultured cells were assessed according to manufacturer's instruction. Absorbance was read by FLUOstar Omega (BMG Labtech.).

### Plaque Assay

Viral titer of mouse tissues or cell lysates was assessed by a plaque assay on NIH 3T3 monolayer essentially as previously described [22]. Briefly, after three rounds of freezing and thawing, 10-fold serially-diluted virus-containing supernatant was added onto NIH 3T3 cells and incubated for 2 hours at 37°C. Then, DMEM containing 2% NCS and 0.75% methylcellulose (Sigma) was added after removing the supernatant. Plaques were counted at day 6 post-infection. The detection limit for this assay is 5 PFU. To assess the antiviral effect of IL6 and TNFα, wild-type MEFs were plated at an initial cell density of 5,000 cells/cm^2^, and infected with γHV68 at a multiplicity-of-infection (MOI) of 0.005. DMEM containing 2 ng/ml rmIL6 or rmTNFα were added to cells at 2 hours before infection. Medium was removed at 2 hours post-infection, and cells were washed with medium and incubated in DMEM containing 2% FBS and 0.75% methylcellulose. Plaques were counted at day 5 post-infection.

### Histology

*Mavs* ^+/+^ and *Mavs* ^−/−^ littermate mice were intranasally infected with 40 PFU of γHV68 as described above. Mouse right lungs were fixed in the neutral buffered 10% formalin solution (Sigma) overnight at 4°C. Tissue specimens were dehydrated, embedded in paraffin, and cut into 3 mm sections. Lung sections were analyzed by hematoxylin and eosin (H&E), immunohistochemistry, and cytochemistry staining. Macrophages were stained with rabbit anti-Iba1 polyclonal antibody (Wako), rabbit ABC staining system (Santa Cruz), and DAB substrate kit (Vector laboratories). Neutrophils were stained with the Naphthol AS-D Chloroacetate Specific Esterase Kit (Sigma). Hematoxylin solutions for countertaining include: Gril No. 2 for macrophage staining, and Gril No. 3 for neutrophil staining.

### Reverse Transcription (RT)-PCR and Quantitative Real-Time PCR (qRT-PCR)

To determine the relative levels of cytokine transcripts, RT-PCR and qRT-PCR were performed as previously reported [22]. Briefly, total RNA was extracted from MEFs or mouse tissues using TRIzol reagent (Invitrogen). To remove genomic DNA, total RNA was digested with RNase-free DNase I (New England Biolab) at 37°C for 1 hour. After heat inactivation, total RNA was re-purified with TRIzol reagent. cDNA was prepared with 1.5 µg total RNA and reverse transcriptase (Invitrogen). RNA was then removed by incubation with RNase H (Epicentre). The abundance of cytokine mRNAs and viral transcripts was assessed by qRT-PCR using 7500 Fast Real-Time PCR system (Applied Biosystems). Mouse β-actin was used as an internal control. All primers were designed by Primer Express 3.0 (Applied Biosystems) and validated individually (Table S1).

### Nuclear Extraction and Electrophoresis Mobility Shift Assay (EMSA)

MEFs were infected with γHV68 K3/GFP (MOI = 5), and harvested at indicated time points. Cells were washed once with ice-cold PBS, scrapped into 5 ml cold PBS on ice, and centrifuged at 2,000 g, 4°C for 5 min. Cell pellets were resuspended in ice-cold hypotonic lysis buffer (10 mM Tris-HCl [pH 7.4], 150 mM NaCl, 1.5 mM MgCl_2_, 0.5 mM phenylmethylsulfonyl fluoride, 10 mM dithiothreitol, 0.65% Nonidet P-40). Nuclei were spun down and rinsed with ice-cold hypotonic lysis buffer without Nonidet P-40. Nuclei were resuspended in a low salt buffer (20 mM HEPES [pH 7.9], 2 mM EDTA [pH 8.0], 20 mM KCl, 1.5 mM MgCl_2_, 0.5 mM phenylmethylsulfonyl fluoride, 0.5 mM dithiothreitol, 25% glycerol). Then, a high salt buffer (20 mM HEPES [pH 7.9], 2 mM EDTA [pH 8.0], 800 mM KCl, 1.5 mM MgCl_2_, 0.5 mM phenylmethylsulfonyl fluoride, 0.5 mM dithiothreitol, 25% glycerol) was added in a dropwise fashion while stirring gently. The supernatant (nuclear extract) was collected by centrifugation at 25,000 g for 30 min at 4°C.

Nuclear extracts were analyzed for NFκB activation by EMSA. Two micrograms of nuclear extracts were incubated with a ^32^P-labeled oligonucleotide (Promega) containing the NFκB consensus site (5′-AGT TGA GGG GAC TTT CCC AGG C-3′) for 15 minutes at room temperature in a binding reaction containing 10 mM Tris-HCl (pH 7.5), 0.5 mM EDTA, 50 mM NaCl, 1 mM MgCl_2,_ 0.5 mM dithiothreitol, 0.05 mg/ml poly(dI-dC) (Sigma), 4% glycerol. For the competition assay and the super-shift assay, 50-fold molar excess of cold probe or 20 µg/ml (final concentration) mouse monoclonal anti-RelA (sc-8008, Santa Cruz Biotech.) was separately pre-incubated with nuclear extracts for 10 min before adding the ^32^P-labeled probe. DNA-protein complexes were subjected to electrophoresis in 6% native polyacrylamide gels (0.25 × TBE) at a constant current of 9 mA. Gels were dried and analyzed by STORM 820 (Amersham Bioscience) for autoradiography.

### Establishing Stable Cell Lines

Lentivirus production in 293T cells was carried out as previously described [22]. Briefly, 293T cells were co-transfected with the packaging plasmids (VSV-G and DR8.9) and pCDH derived plasmids expressing Flag-tagged IκBαΔN, wild-type RelA, or the S468A RelA variant carrying the Serine 468-to-Alanine mutation. At 72 hours post-transfection, supernatant was collected and passed through 0.45 mm filter. Mouse embryonic fibroblasts (MEFs) were infected with filtered lentivirus in complete DMEM containing 10 µg/ml polybrene. To maximize the infection efficiency, cells were centrifuged at 1,800 rpm, 30°C for 1 hour, and incubated at 37°C up to 6 hours. MEFs were selected and maintained in complete DMEM containing 1 µg/ml puromycin.

### Confocal Microscopy

To assess RelA nuclear translocation, control wild-type MEFs or those stably expressing the Flag-tagged IκBαΔN were treated with 10 ng/ml TNFα for 30 minutes, or infected with γHV68 K3/GFP (MOI = 5). Thirty minutes later, γHV68-infected cells were treated with 20 µM MG132 for 2 hours or left untreated. Cells were fixed, permeabilized, stained with rabbit anti-RelA antibody and Alex 596-congugated goat anti-rabbit secondary antibody, and analyzed with confocal microscope (Leica).

### Immunoprecipitation and Immunoblot

Immunoprecipitation and immunoblot were as previously described [22], [55]. Briefly, cells were harvested, rinsed once with ice-cold PBS, and resuspended with RIPA buffer (50 mM Tris-HCl [pH 7.4], 150 mM NaCl, 0.5% sodium deoxycholate, 0.1% SDS, 1% NP40, 5 mM EDTA/EGTA) supplemented with protease inhibitor cocktail. Centrifuged supernatant was pre-cleared with protein A/G agarose at 4°C for one hour, and subjected to precipitation by incubating with anti-RelA antibody and protein A/G agarose, or anti-Flag M2-conjugated agarose. Precipitated proteins were extensively washed with RIPA buffer and eluted with 1 × SDS-PAGE loading buffer by boiling at 95°C for 5 - 10 min.

For immunoblot analysis, WCLs (20 µg) or precipitated proteins were resolved by SDS-PAGE, and transferred to PVDF membrane. Immunoblot detection was performed with corresponding primary antibodies as indicated by incubating at 4°C overnight and with secondary peroxidase-conjugated antibody for one hour. Proteins were visualized with SuperSignal West Pico Chemiluminescent Substrate (Thermo Scientific) and a Fujifilm LAS-3000 imaging system (FujiFilm).

### Antibodies

Commercial antibodies used in this study include: anti-Flag (Sigma), anti-IκBα (sc-371, Santa Cruz Biotech.), rabbit anti-RelA (sc-372-G, Santa Cruz Biotech.), mouse anti-RelA (sc-8008, Santa Cruz Biotech.), anti-RelA S536p (93H1, Cell Signaling), anti-RelA S468p (Bethyl Group), anti-β-actin (Abcam.), anti-ubiquitin-conjugated protein (FK2, Affiniti Research Products), and anti-Iba1 (Wako).

### Statistical Analysis

The statistical significance (*P*-value) is calculated by unpaired two-tailed Student's *t*-test. *, *P*<0.05; **, *P*<0.02; ***, *P*<0.005. A *P*-value of <0.05 is considered statistically significant.

### NCBI Entrez Gene ID (*Mus musculus*) List

RIG-I, 230073; MDA-5, 71586; MAVS, 228607; TBK1, 56480; IKKε, 56489; IRF3, 54131; IRF7, 54123; c-Jun, 16476; ATF-2, 11909; p300, 328572; IFNβ, 15977; IKKγ, 16151; IKKα, 12675; IKKβ, 16150; IκBα, 18035; NFκB1, 18033; RelA, 19697; NFκB2, 18034; RelB, 19698; c-Rel, 19696; IL6, 16193; TNFα, 21926; CCL5, 20304; CXCL1, 14825; IL10, 16153.

## Supporting Information

Figure S1 **Cytokine levels of** **γHV68-infected** ***Mavs*** **^+/+^ and** ***Mavs*** **^−/−^ littermates.** Age- and gender-matched *Mavs* ^+/+^ and *Mavs* ^−/−^ littermate mice (eight mice per group) were intranasally infected with 40 plaque-forming units (PFU) of γHV68. Cytokine levels in the serum (A) or the lung (B) of γHV68-infected mice were determined by ELISA. (A) There was no significant difference of serum cytokines (except CCL5 at 10 and 16 days post-infection) between *Mavs* ^+/+^ and *Mavs* ^−/−^ littermates. (B) There was no significant difference of anti-inflammatory cytokine IL10 in the lung between *Mavs* ^+/+^ and *Mavs* ^−/−^ littermates. Data are presented as the mean ± the standard error of the mean (SEM) of eight mice. The statistical significance: **, *P*<0.02.
(PDF)

Figure S2 **Intranasal administration using a total volume of 30 µl efficiently delivers protein into mouse lung**. To assess the delivery efficiency of protein, buffer (1% BSA in PBS) alone and firefly luciferase diluted in 15 µl or 30 µl buffer were intranasally administered to BL6 mice (five to six mice per group). (A) Mouse tissues (nasal cavity, trachea, and lung) were harvested and homogenized at 2 hours post administration. (B) Relative luciferase activity in the trachea and the lung was normalized to that in the nasal cavity. Each symbol represents one mouse. The statistical significance: ***, *P*<0.005. (C) The distribution percentage of firefly luciferase among the nasal cavity, trachea, and lung. Data are presented as the mean ± SEM.
(PDF)

Figure S3 **Intranasal administration of recombinant mouse IL6 or TNFα** **does not affect mouse health.** Age- and gender-matched BL/6 mice were intranasally infected with 40 PFU of γHV68 (eight mice per group). Buffer (1% BSA in PBS) alone, recombinant mouse IL6 or TNFα (rmIL6 or rmTNFα, 30 ng/mouse/day) were intranasally administered from 1 to 5 days post-infection (d.p.i.). All mice were sacrificed at 6 d.p.i. (A) Body weight of all mice was recorded during the entire experimental period. Each cross (×) represents one mouse. There was no significant gain or loss of the body weight among the mock-,rmIL6-, or rmTNFα-treated mice. (B) Spleen mass was measured at 6 d.p.i. There was no significant difference of spleen size or weight among the mock-, rmIL6-, or rmTNFα-treated mice. (C and D) IL6 and TNFα levels in the lung at 6 d.p.i. were determined by ELSIA. Data in (B), (C) and (D) are presented as the mean ± SEM of eight mice. The statistical significance: **, *P*<0.02.
(PDF)

Figure S4 **Deficiency in MAVS results in an elevated immune cell infiltration in the lung of** **γHV68-infected mice.** Age- and gender-matched *Mavs* ^+/+^ and *Mavs* ^−/−^ littermate mice were intranasally infected with 40 PFU of γHV68. At 10 days post-infection, mouse lungs were fixed and embedded in paraffin. Three-micrometer sections were analyzed by hematoxylin and eosin (H&E) staining (A), immunohistochemistry staining (B and C), and cytochemistry staining (D). Pictures were taken at the magnification of 200. One to three optical fields are presented for each group. (A) H&E staining of paraffin sections demonstrated a mild mixed-cell infiltration (lymphocytes and macrophages dominant, and neutrophils rare) causing diffuse increased interstitial cellularity (*black arrowhead*) in the lungs of γHV68-infected *Mavs* ^+/+^ mice. In the lungs of γHV68-infected *Mavs* ^−/−^ mice, there was an intense peribronchial and perivascular immune infiltration (*black arrow*). A, airway; V, blood vessel. (B and C) Pulmonary macrophages were probed with anti-Iba1 antibody. (B) The negative control was set up for all sections. One representative positively stained optical field was shown in comparison to its negative control. (C) γHV68 infection induced more peribronchial and perivascular macrophage infiltrates in *Mavs* ^−/−^ mice than those in *Mavs* ^+/+^ mice. (D) Pulmonary neutrophils (*red arrowheads*) were selectively stained by an esterase specific assay. γHV68 infection induced a significant increase of perivascular neutrophils in the lungs of *Mavs* ^−/−^ mice, but not in those of *Mavs* ^+/+^ mice.
(PDF)

Figure S5 **Deficiency in MAVS impairs Sendai virus (SeV)-induced cytokine production by bone marrow-derived macrophages (BMDMs).** BMDMs from *Mavs* ^+/+^ and *Mavs* ^−/−^ littermate mice were infected with 500 HA units of Sendai virus for 12 hours. Cytokine levels in the supernatant were determined by ELISA. Data are presented as the mean ± SEM of three independent experiments. The statistical significance: **, *P*<0.02; ***, *P*<0.005.
(PDF)

Figure S6 **γHV68 slightly reduces cytokine levels in the lung during early infection** ***in vivo*** **.** Age- and gender-matched BL6 mice (five mice per group) were intranasally infected with 1 × 10^5^ PFU of γHV68. Cytokine Levels (IL6, TNFα, CCL5 and CXCL1) in the lungs of mock- or γHV68-infected BL6 mice at 2.5 days post-infection were determined by ELISA (A) or quantitative real-time PCR (qRT-PCR) using β-actin as an internal control (B). Each symbol represents one mouse. (A) γHV68 infection slightly reduced cytokine levels in the lung. The statistical significance: *, *P*<0.05. (B) To quantify viral lytic replication in the lung of γHV68-infected mice, RTA mRNA levels were determined by qRT-PCR. Red dashed lines represent the trend lines of regression between mRNA levels of cytokines and those of γHV68 RTA. The *r* stands for Pearson product-moment correlation coefficient between mRNA levels of cytokines and those of γHV68 RTA.
(PDF)

Figure S7 **MAVS deficiency impairs** γ**HV68-induced IFNβ** **expression in mouse embryonic fibroblasts (MEFs).** *Mavs* ^+/+^ and *Mavs* ^−/−^ MEFs were infected with γHV68 at an MOI of 5. Cells were collected at indicated time points, and IFNβ mRNA levels were determined by real-time PCR using β-actin as an internal control. Data are presented as the mean ± SEM of three independent experiments. The statistical significance: *, *P*<0.05; **, *P*<0.02.
(PDF)

Figure S8 **γHV68 fails to induce cytokine response in mouse embryonic fibroblasts (MEFs) deficient in IKKβ** **or IKKγ** **.** See also Figure 4B. Wild-type MEFs, or those deficient in MAVS, IKKβ or IKKγ were infected with γHV68 (MOI = 5). Relative quantity of cytokine mRNAs in γHV68-infected MEFs were analyzed by real-time PCR and normalized to that of β-actin.
(PDF)

Figure S9 **NFκ** **B activation in** ***Mavs*** **^+/+^ and** ***Mavs*** **^−/−^ mouse embryonic fibroblasts (MEFs) by electrophoresis mobility shift assay.** *Mavs* ^+/+^ and *Mavs* ^−/−^ MEFs were incubated with buffer alone (negative control) or 10 ng/ml TNFα for 30 minutes (positive control). Nuclear extracts (2 µg) were subjected to electrophoresis mobility shift assay using a [^32^P]-NFκB probe, without or with pre-incubating with cold NFκB probe or a monoclonal anti-RelA antibody.
(PDF)

Figure S10 **Expression of Iκ** **BαΔ** **N blocks nuclear translocation of RelA.** Wild-type mouse embryonic fibroblasts (MEFs) stably expressing the Flag-tagged IκBα super-suppressor (IκBαΔN) were established as described in Materials and Methods. MEFs were treated with 10 ng/ml TNFα, fixed, and permeabilized. Cells were stained with rabbit anti-RelA antibody and Alex 596-congugated goat anti-rabbit secondary antibody, analyzed with confocal microscope (Leica). A representative field was shown for each time point. The percentage of cells showing RelA nuclear translocation was calculated based on 200 cells.
(PDF)

Figure S11 **IKKα** **deficiency does not impair** **γHV68-induced RelA degradation.** Wild-type and IKKα-deficient MEFs were infected with γHV68 at an MOI of 20 and cells were harvested at indicated time points. Whole cell lysates were analyzed by immunoblot with antibodies to RelA and actin.
(PDF)

Figure S12 **Expression of RelA.S468A variant impairsγ** **HV68 lytic replication in mouse embryonic fibroblasts (MEFs).** Control (Vector), and wild-type MEFs expressing RelA.S468A-expressing or IκBαΔN were infected with γHV68 at a multiplicity-of-infection (MOI) of 0.01 (A) or 0.005 (B). (A) Viral titer in the supernatant collected at 1 d.p.i. and 2 d.p.i. was determined by a plaque assay. (B) At 2 hours post-infection, supernatant was replaced with fresh DMEM containing 2% FBS and 0.75% methylcellulose. Plaques formed in MEF monolayers were counted at 6 d.p.i. Data in (A) and (B) are presented as the mean ± SEM of three independent experiments. The statistical significance: *, *P*<0.05.
(PDF)

Table S1 **Primer list.** PCR primers were designed by MacVector 9.0 (Accelrys Inc.), and quantitative real-time PCR (qRT-PCR) primers were designed by Primer Express 3.0 (Applied Biosystems). All primers used in this study were synthesized by Invitrogen.
(PDF)
