# Supplementary figures and images for: Murine Gamma Herpesvirus 68 Hijacks MAVS and IKKβ to Abrogate NFκB Activation and Antiviral Cytokine Production
Source: PLoS Pathog. 2011 Nov 10;7(11):e1002336. doi: 10.1371/journal.ppat.1002336 (PMC3213086; doi:10.1371/journal.ppat.1002336)

# Figure S1

A

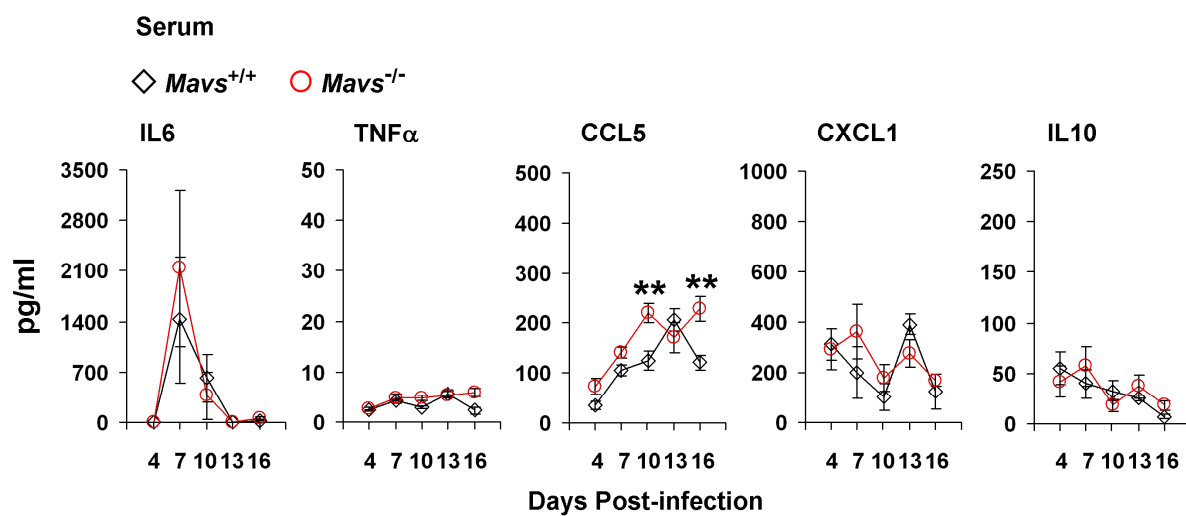

B

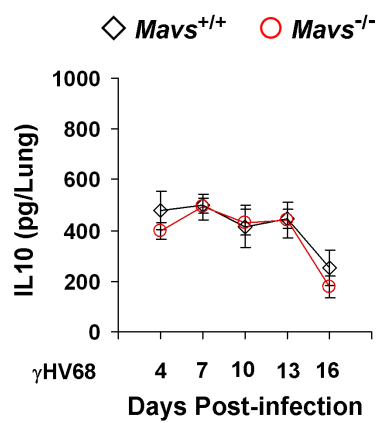

Supplement: Figure S1 — Cytokine levels of γHV68-infected Mavs +/+ and Mavs −/− littermates. Age- and gender-matched Mavs +/+ and Mavs −/− littermate mice (eight mice per group) were intranasally infected with 40 plaque-forming units (PFU) of γHV68. Cytokine levels in the serum (A) or the lung (B) of γHV68-infected mice were determined by ELISA. (A) There was no significant difference of serum cytokines (except CCL5 at 10 and 16 days post-infection) between Mavs +/+ and Mavs −/− littermates. (B) There was no significant difference of anti-inflammatory cytokine IL10 in the lung between Mavs +/+ and Mavs −/− littermates. Data are presented as the mean ± the standard error of the mean (SEM) of eight mice. The statistical significance: **, P<0.02. (PDF) [file ppat.1002336.s001.pdf]

# Figure S2

A

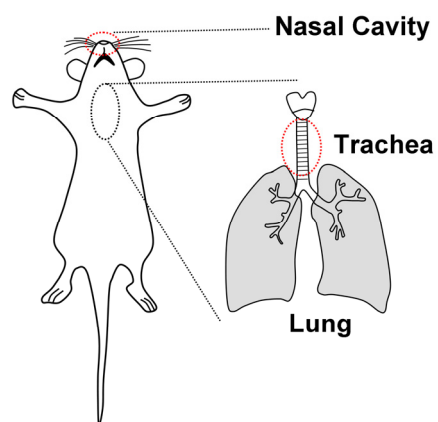

B

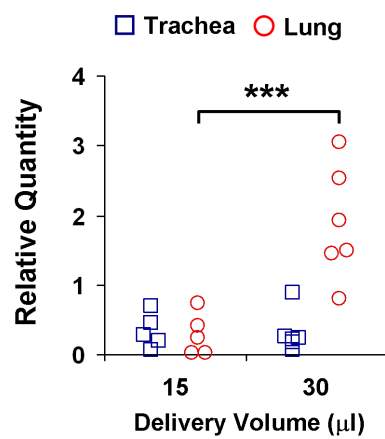

C

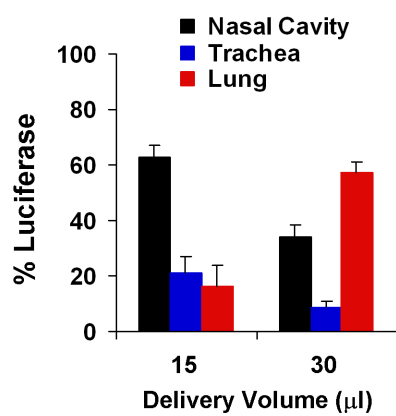

Supplement: Figure S2 — Intranasal administration using a total volume of 30 µl efficiently delivers protein into mouse lung. To assess the delivery efficiency of protein, buffer (1% BSA in PBS) alone and firefly luciferase diluted in 15 µl or 30 µl buffer were intranasally administered to BL6 mice (five to six mice per group). (A) Mouse tissues (nasal cavity, trachea, and lung) were harvested and homogenized at 2 hours post administration. (B) Relative luciferase activity in the trachea and the lung was normalized to that in the nasal cavity. Each symbol represents one mouse. The statistical significance: ***, P<0.005. (C) The distribution percentage of firefly luciferase among the nasal cavity, trachea, and lung. Data are presented as the mean ± SEM. (PDF) [file ppat.1002336.s002.pdf]

# Figure S3

A

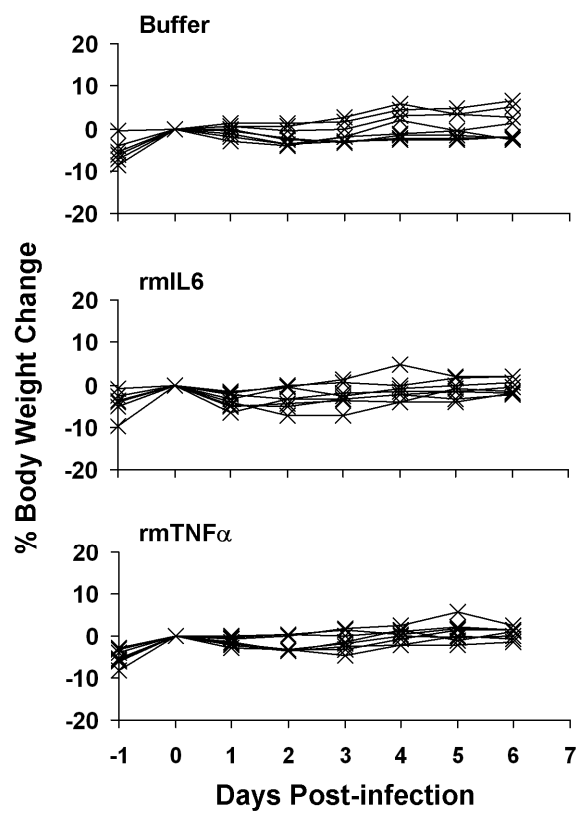

C

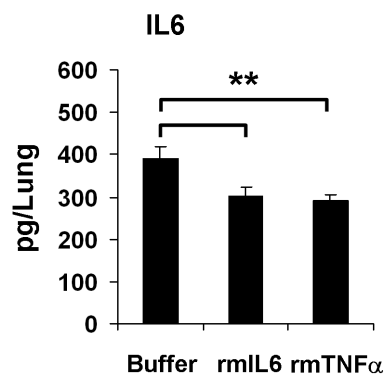

D

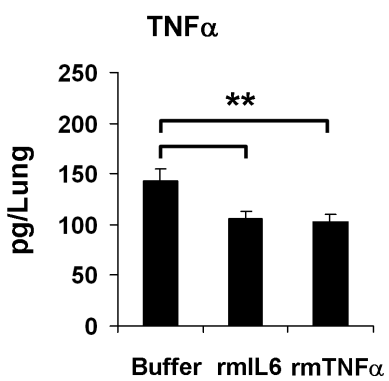

B

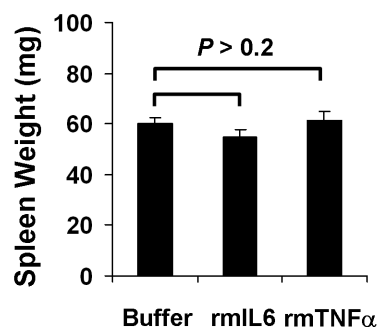

Supplement: Figure S3 — Intranasal administration of recombinant mouse IL6 or TNFα does not affect mouse health. Age- and gender-matched BL/6 mice were intranasally infected with 40 PFU of γHV68 (eight mice per group). Buffer (1% BSA in PBS) alone, recombinant mouse IL6 or TNFα (rmIL6 or rmTNFα, 30 ng/mouse/day) were intranasally administered from 1 to 5 days post-infection (d.p.i.). All mice were sacrificed at 6 d.p.i. (A) Body weight of all mice was recorded during the entire experimental period. Each cross (×) represents one mouse. There was no significant gain or loss of the body weight among the mock-,rmIL6-, or rmTNFα-treated mice. (B) Spleen mass was measured at 6 d.p.i. There was no significant difference of spleen size or weight among the mock-, rmIL6-, or rmTNFα-treated mice. (C and D) IL6 and TNFα levels in the lung at 6 d.p.i. were determined by ELSIA. Data in (B), (C) and (D) are presented as the mean ± SEM of eight mice. The statistical significance: **, P<0.02. (PDF) [file ppat.1002336.s003.pdf]

# Figure S4

**A**

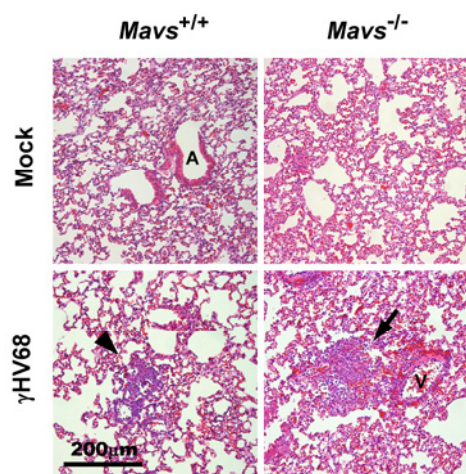

Staining: H&E

**B**

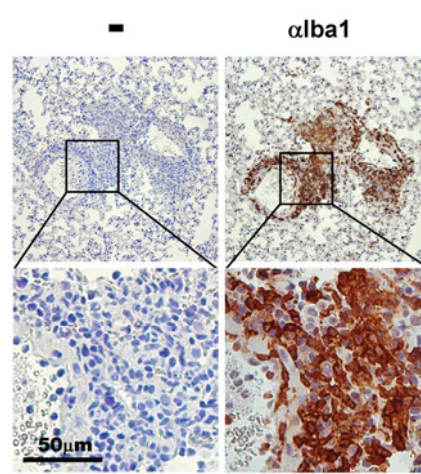

Staining: MΦ

**C**

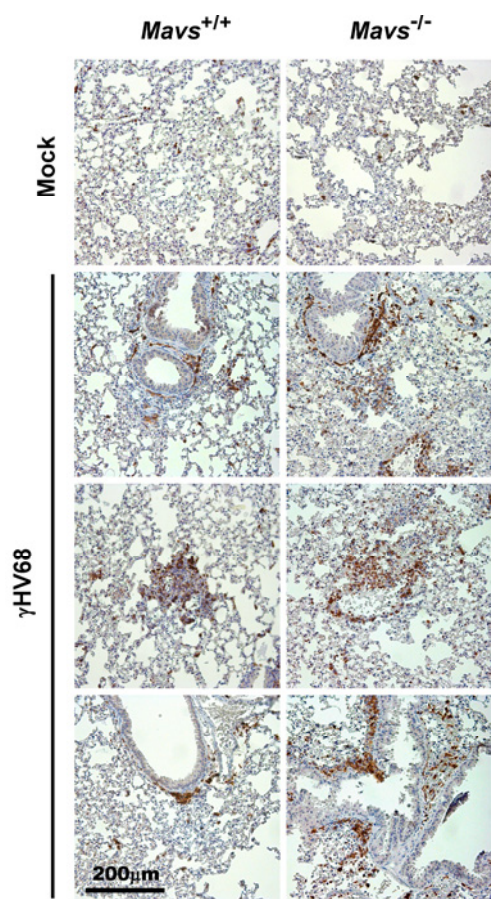

Staining: MΦ

**D**

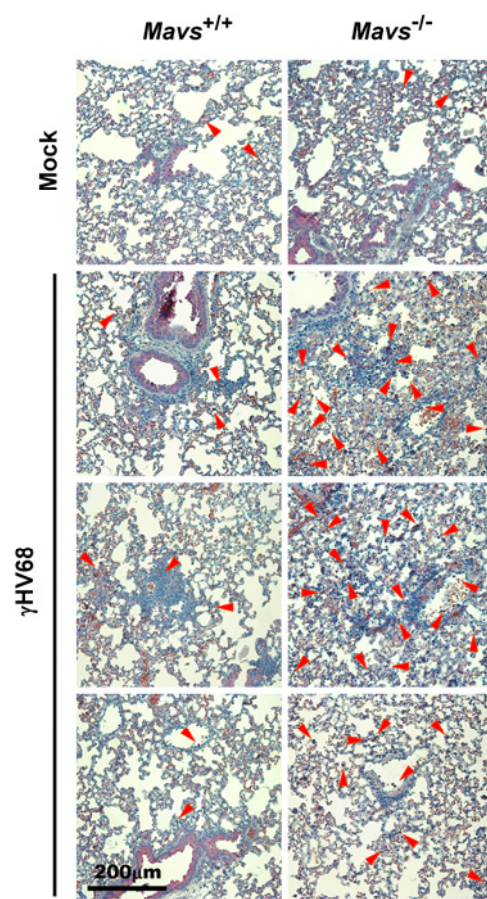

Staining: Neutrophils

Supplement: Figure S4 — Deficiency in MAVS results in an elevated immune cell infiltration in the lung of γHV68-infected mice. Age- and gender-matched Mavs +/+ and Mavs −/− littermate mice were intranasally infected with 40 PFU of γHV68. At 10 days post-infection, mouse lungs were fixed and embedded in paraffin. Three-micrometer sections were analyzed by hematoxylin and eosin (H&E) staining (A), immunohistochemistry staining (B and C), and cytochemistry staining (D). Pictures were taken at the magnification of 200. One to three optical fields are presented for each group. (A) H&E staining of paraffin sections demonstrated a mild mixed-cell infiltration (lymphocytes and macrophages dominant, and neutrophils rare) causing diffuse increased interstitial cellularity (black arrowhead) in the lungs of γHV68-infected Mavs +/+ mice. In the lungs of γHV68-infected Mavs −/− mice, there was an intense peribronchial and perivascular immune infiltration (black arrow). A, airway; V, blood vessel. (B and C) Pulmonary macrophages were probed with anti-Iba1 antibody. (B) The negative control was set up for all sections. One representative positively stained optical field was shown in comparison to its negative control. (C) γHV68 infection induced more peribronchial and perivascular macrophage infiltrates in Mavs −/− mice than those in Mavs +/+ mice. (D) Pulmonary neutrophils (red arrowheads) were selectively stained by an esterase specific assay. γHV68 infection induced a significant increase of perivascular neutrophils in the lungs of Mavs −/− mice, but not in those of Mavs +/+ mice. (PDF) [file ppat.1002336.s004.pdf]

**Figure S5**

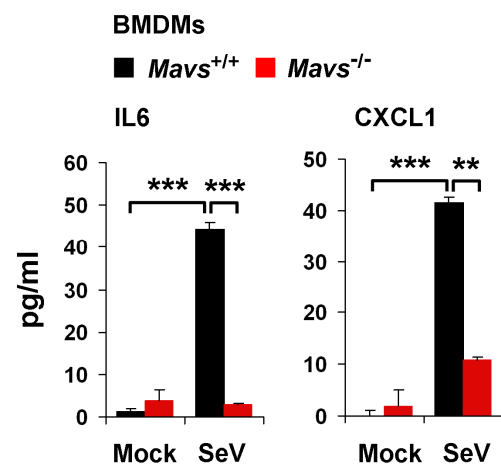

Supplement: Figure S5 — Deficiency in MAVS impairs Sendai virus (SeV)-induced cytokine production by bone marrow-derived macrophages (BMDMs). BMDMs from Mavs +/+ and Mavs −/− littermate mice were infected with 500 HA units of Sendai virus for 12 hours. Cytokine levels in the supernatant were determined by ELISA. Data are presented as the mean ± SEM of three independent experiments. The statistical significance: **, P<0.02; ***, P<0.005. (PDF) [file ppat.1002336.s005.pdf]

# Figure S6

A

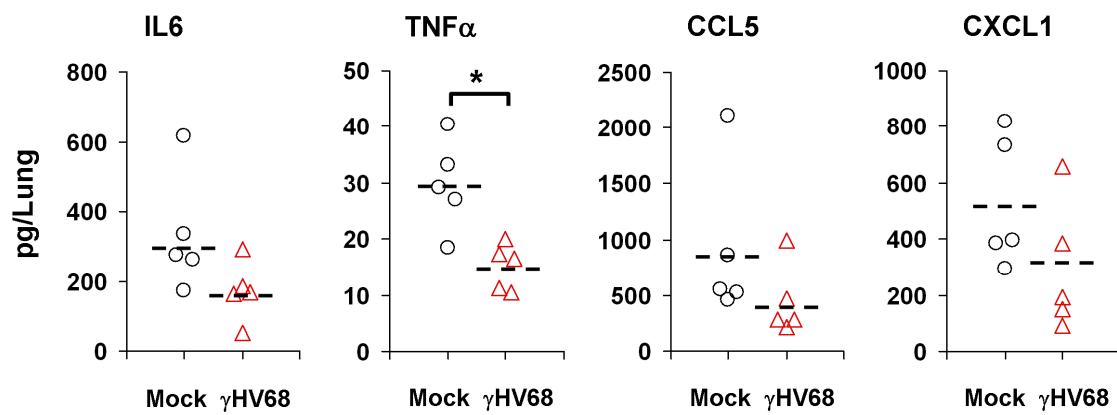

B

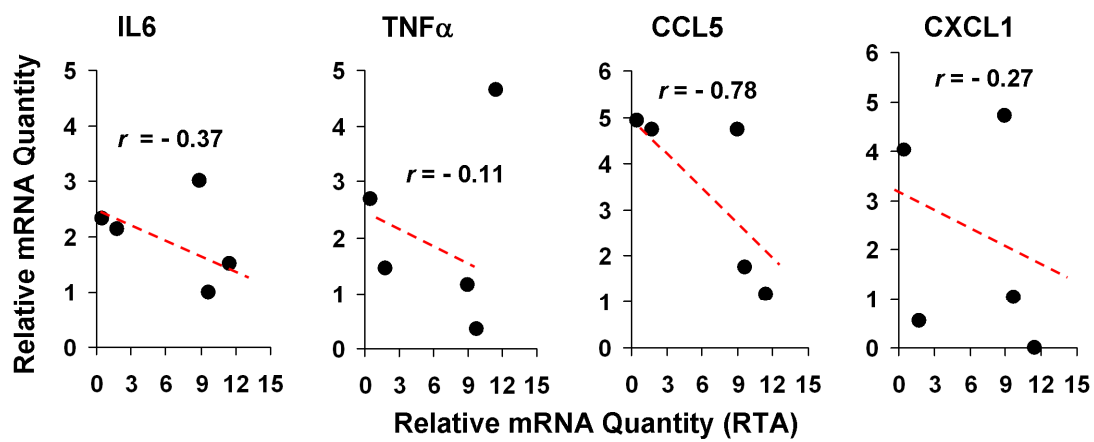

Supplement: Figure S6 — γHV68 slightly reduces cytokine levels in the lung during early infection in vivo . Age- and gender-matched BL6 mice (five mice per group) were intranasally infected with 1 × 10^5^ PFU of γHV68. Cytokine Levels (IL6, TNFα, CCL5 and CXCL1) in the lungs of mock- or γHV68-infected BL6 mice at 2.5 days post-infection were determined by ELISA (A) or quantitative real-time PCR (qRT-PCR) using β-actin as an internal control (B). Each symbol represents one mouse. (A) γHV68 infection slightly reduced cytokine levels in the lung. The statistical significance: *, P<0.05. (B) To quantify viral lytic replication in the lung of γHV68-infected mice, RTA mRNA levels were determined by qRT-PCR. Red dashed lines represent the trend lines of regression between mRNA levels of cytokines and those of γHV68 RTA. The r stands for Pearson product-moment correlation coefficient between mRNA levels of cytokines and those of γHV68 RTA. (PDF) [file ppat.1002336.s006.pdf]

**Figure S7**

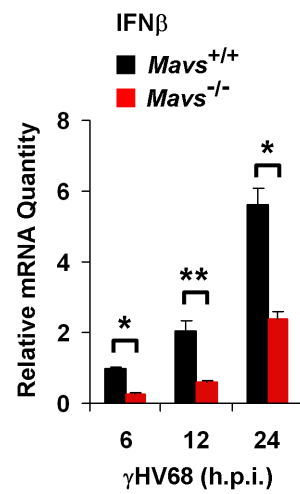

Supplement: Figure S7 — MAVS deficiency impairs γHV68-induced IFNβ expression in mouse embryonic fibroblasts (MEFs). Mavs +/+ and Mavs −/− MEFs were infected with γHV68 at an MOI of 5. Cells were collected at indicated time points, and IFNβ mRNA levels were determined by real-time PCR using β-actin as an internal control. Data are presented as the mean ± SEM of three independent experiments. The statistical significance: *, P<0.05; **, P<0.02. (PDF) [file ppat.1002336.s007.pdf]

Figure S8

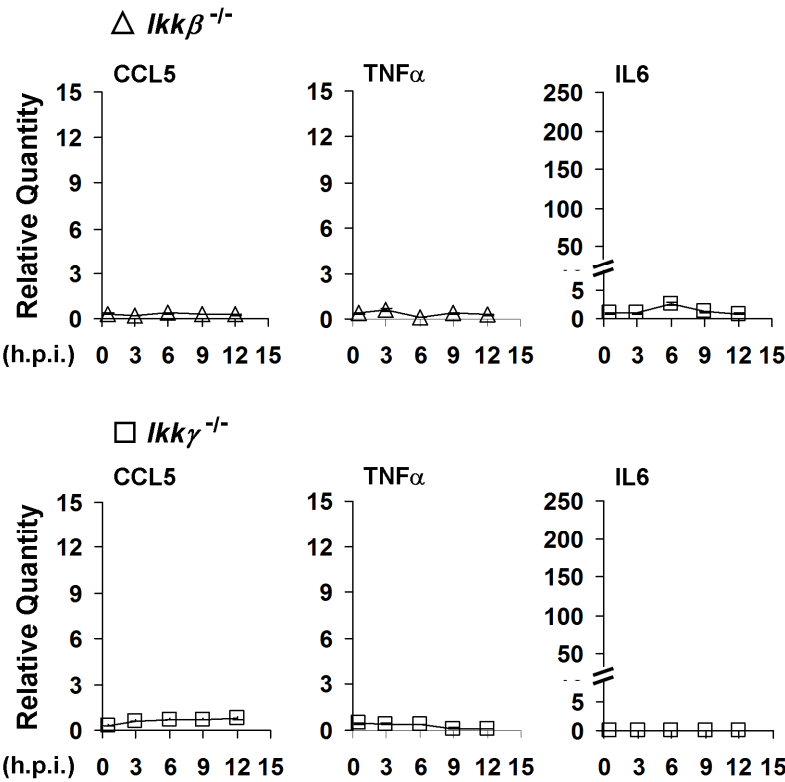

Supplement: Figure S8 — γHV68 fails to induce cytokine response in mouse embryonic fibroblasts (MEFs) deficient in IKKβ or IKKγ . See also Figure 4B. Wild-type MEFs, or those deficient in MAVS, IKKβ or IKKγ were infected with γHV68 (MOI = 5). Relative quantity of cytokine mRNAs in γHV68-infected MEFs were analyzed by real-time PCR and normalized to that of β-actin. (PDF) [file ppat.1002336.s008.pdf]

Figure S9

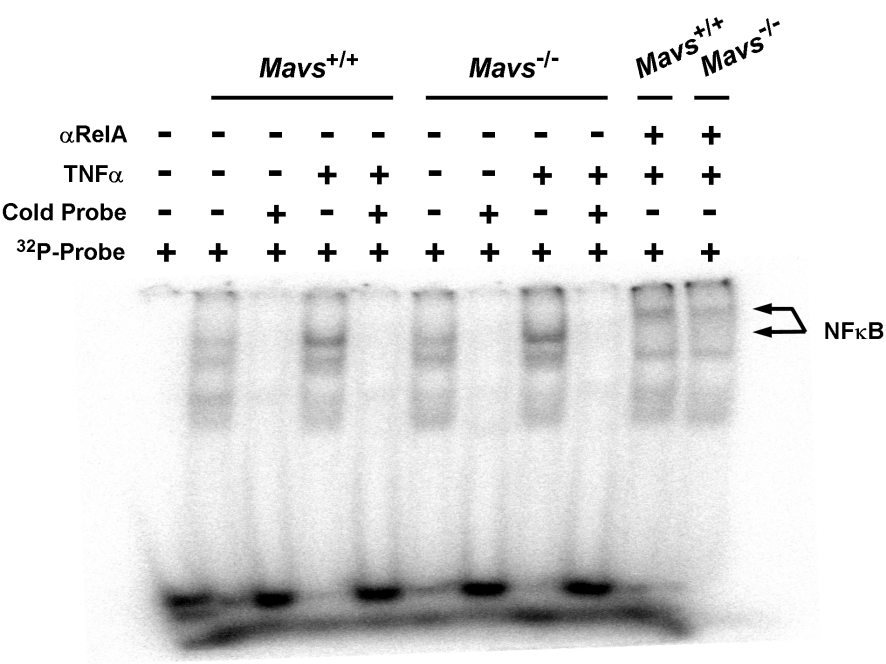

Supplement: Figure S9 — NFκ B activation in Mavs +/+ and Mavs −/− mouse embryonic fibroblasts (MEFs) by electrophoresis mobility shift assay. Mavs +/+ and Mavs −/− MEFs were incubated with buffer alone (negative control) or 10 ng/ml TNFα for 30 minutes (positive control). Nuclear extracts (2 µg) were subjected to electrophoresis mobility shift assay using a [32P]-NFκB probe, without or with pre-incubating with cold NFκB probe or a monoclonal anti-RelA antibody. (PDF) [file ppat.1002336.s009.pdf]

**Figure S10**

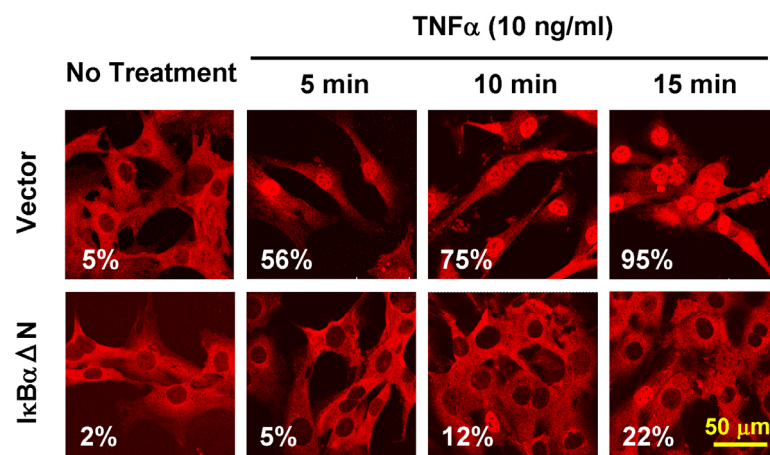

Supplement: Figure S10 — Expression of Iκ BαΔ N blocks nuclear translocation of RelA. Wild-type mouse embryonic fibroblasts (MEFs) stably expressing the Flag-tagged IκBα super-suppressor (IκBαΔN) were established as described in Materials and Methods. MEFs were treated with 10 ng/ml TNFα, fixed, and permeabilized. Cells were stained with rabbit anti-RelA antibody and Alex 596-congugated goat anti-rabbit secondary antibody, analyzed with confocal microscope (Leica). A representative field was shown for each time point. The percentage of cells showing RelA nuclear translocation was calculated based on 200 cells. (PDF) [file ppat.1002336.s010.pdf]

Figure S11

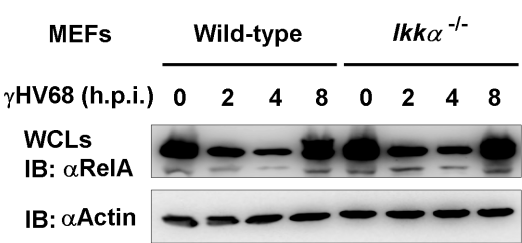

Supplement: Figure S11 — IKKα deficiency does not impair γHV68-induced RelA degradation. Wild-type and IKKα-deficient MEFs were infected with γHV68 at an MOI of 20 and cells were harvested at indicated time points. Whole cell lysates were analyzed by immunoblot with antibodies to RelA and actin. (PDF) [file ppat.1002336.s011.pdf]

Figure S12

A

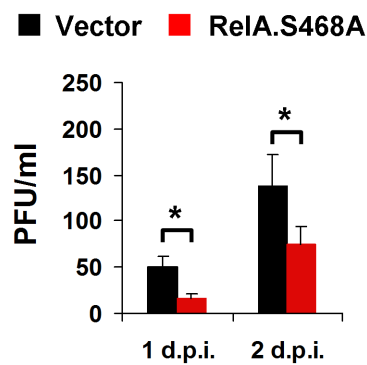

B

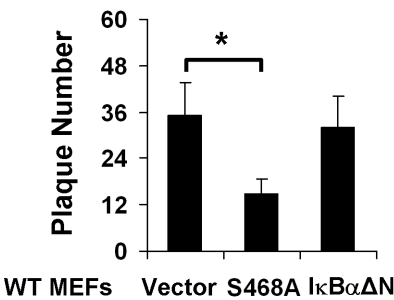

Supplement: Figure S12 — Expression of RelA.S468A variant impairsγ HV68 lytic replication in mouse embryonic fibroblasts (MEFs). Control (Vector), and wild-type MEFs expressing RelA.S468A-expressing or IκBαΔN were infected with γHV68 at a multiplicity-of-infection (MOI) of 0.01 (A) or 0.005 (B). (A) Viral titer in the supernatant collected at 1 d.p.i. and 2 d.p.i. was determined by a plaque assay. (B) At 2 hours post-infection, supernatant was replaced with fresh DMEM containing 2% FBS and 0.75% methylcellulose. Plaques formed in MEF monolayers were counted at 6 d.p.i. Data in (A) and (B) are presented as the mean ± SEM of three independent experiments. The statistical significance: *, P<0.05. (PDF) [file ppat.1002336.s012.pdf]
