# Supplementary material for: Murine Gamma Herpesvirus 68 Hijacks MAVS and IKKβ to Abrogate NFκB Activation and Antiviral Cytokine Production
Source: PLoS Pathog. 2011 Nov 10;7(11):e1002336. doi: 10.1371/journal.ppat.1002336 (PMC3213086; doi:10.1371/journal.ppat.1002336)
Supplement: Table S1 — Primer list. PCR primers were designed by MacVector 9.0 (Accelrys Inc.), and quantitative real-time PCR (qRT-PCR) primers were designed by Primer Express 3.0 (Applied Biosystems). All primers used in this study were synthesized by Invitrogen. (PDF) [file ppat.1002336.s013.pdf]

Table S1. Primer List

Primer No.	Description	F or B [1]	Sequence (5'→3')
**D_001**	Murine MAVS genotyping	F	AGCAAGATTCTAGAAGCTGAGAA
**D_002**	Murine MAVS genotyping	B	TAGCTGTGAGGCAGGACAGGTAAGG
**D_003**	Murine MAVS genotyping	B	GTGGAATGTGTGCGCGAGGCCAGAGGC
**D_004**	Reverse Transcription	B	Oligo(dT)$_{12\text{-}19}$
**D_019**	Murine β-Actin qRT-PCR	F	ACGGCCAGGTCATCACTATTG.
**D_020**	Murine β-Actin qRT-PCR	B	CAAGAAGGAAGGCTGGAAAAGA
**D_027**	γHV68 ORF73 qRT-PCR	F	CAAAAACCGCATAATCCATCT
**D_028**	γHV68 ORF73 qRT-PCR	B	CCTGCAGGTGTCTTCGCATT
**D_031**	Murine IL6 qRT-PCR	F	TCCATCCAGTTGCCTTCTTG
**D_032**	Murine IL6 qRT-PCR	B	GGTCTGTTGGGAGTGGTATC
**D_033**	Murine TNFα qRT-PCR	F	CCTCCCTCTCATCAGTTCTATGG
**D_034**	Murine TNFα qRT-PCR	B	GGCTACAGGCTTGTCACTCG
**D_035**	Murine CCL5 qRT-PCR	F	CCTGCTGCTTTGCCTACCTCTC
**D_036**	Murine CCL5 qRT-PCR	B	ACACACTTGGCGGTTCCTTCGA
**D_037**	Murine IFNβ qRT-PCR	F	TCCGAGCAGAGATCTTCAGGAA
**D_038**	Murine IFNβ qRT-PCR	B	TGCAACCACCACTCATTCTGAG

1. F or B: F, Forward primer; B, Backward primer.
